# Upstream migration capacity of *Acrossocheilus fasciatus*: Behavioral strategies in response to hydraulic conditions and implications for low-head weir design

**DOI:** 10.1371/journal.pone.0350692

**Published:** 2026-05-29

**Authors:** Bin Wang, Gang Xu, Lei Fu, Aiju You, Zeqi Xu, Yue Ling

**Affiliations:** 1 River and Lake Research Institute, Zhejiang Institute of Hydraulics and Estuary (Zhejiang Institute of Marine Planning and Design), Hangzhou, Zhejiang Province, China; 2 College of Water Resources and Hydropower Engineering, Hohai University, Nanjing, Jiangsu Province, China; Charles University: Univerzita Karlova, CZECHIA

## Abstract

Current fishway design guidelines are mostly derived from large anadromous fish species, with scarce species-specific data for small-bodied stream cyprinids such as *Acrossocheilus fasciatus*. This study investigated the upstream passage capacity of the cyprinid fish *A. fasciatus* over low-head weirs in relation to body length through controlled flume experiments. We quantified the passage success rate (PSR), behavioral strategies, and key kinematic metrics of upstream movement. Results showed that, under the specific experimental conditions, passage performance and strategy usage pattern were significantly associated with body length. Trial groups with larger mean body length showed a higher proportion of pure leaping strategy (PLS), mean body length per trial group exhibited an extremely strong positive linear relationship with mean leap distance (*R*² = 0.96, *p* < 0.001). Conversely, groups with smaller mean body length showed a higher proportion of Leaping-Swimming Strategy (LSS) usage. The association between body length and leap height was weaker. Under the specific hydraulic and structural conditions tested in this study, we observed the following trends: a weir drop of 25–30 cm allowed partial passage of *A. fasciatus*; the fixed 45° sloped crest profile used in this experiment was associated with prevalent adoption of the LSS by small-bodied individuals (≤13 cm); a downstream pool depth >30 cm and an inter-weir pool length >50 cm were sufficient to accommodate the observed leaping kinematics. These findings provide observational data from a single laboratory configuration for understanding the passage-related bio-behavioral characteristics of small-bodied mountain stream fishes such as *A. fasciatus*. All values reported are preliminary observations under controlled conditions, and their potential relevance to engineering design requires validation through multi-condition comparative experiments and field monitoring.

## Introduction

**Fig 1:**
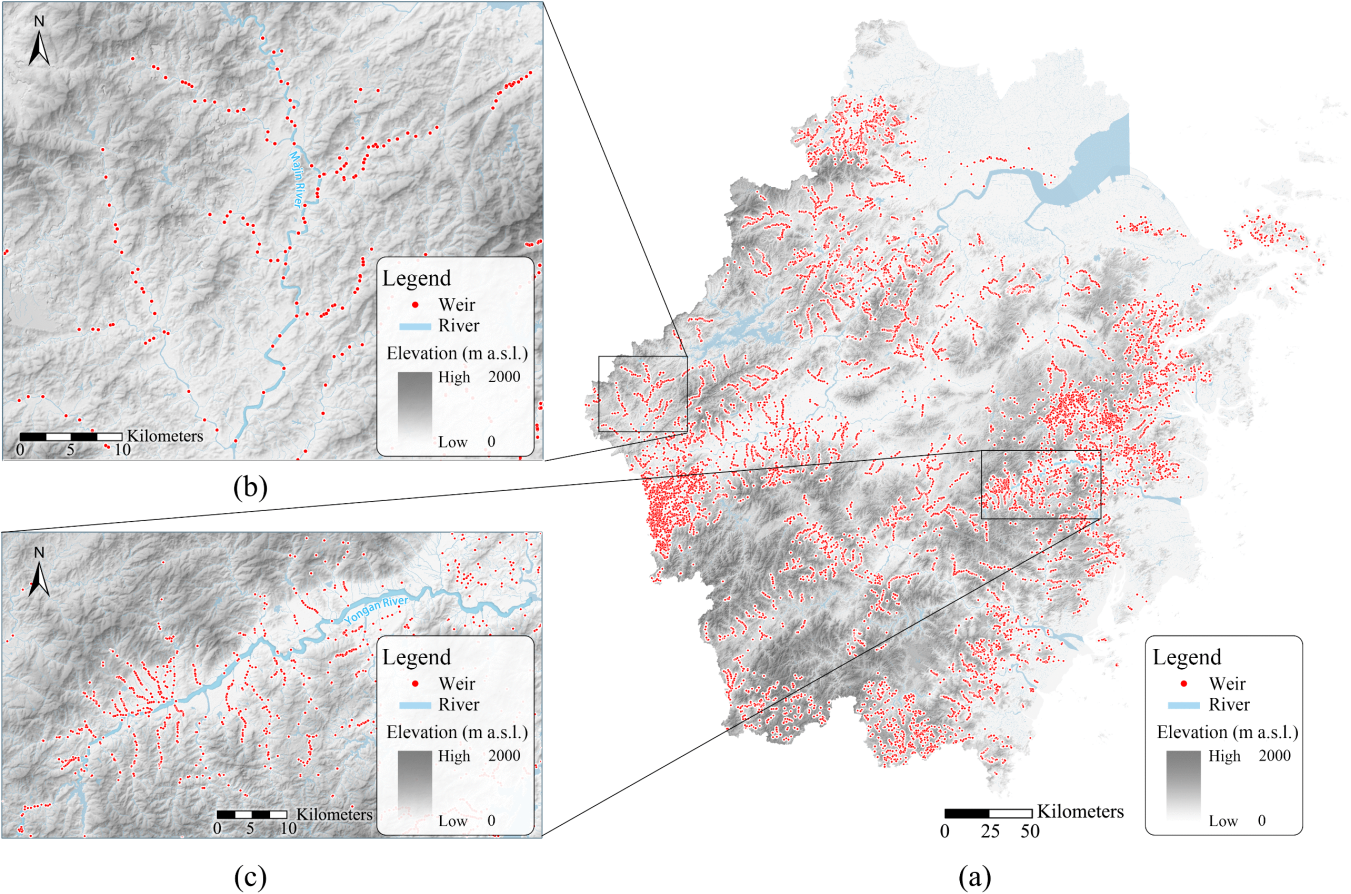
Distribution of Weirs in Zhejiang Province, China. **(a)** Provincial-scale map; **(b)** Majinxi River Basin (the sampling site for experimental fish); **(c)**Yong’anxi River Basin. Base Map Source Attribution: The base maps in this figure were generated using elevation data from the ASTER Global Digital Elevation Model V3 (ASTER GDEM v3; NASA/METI, https://doi.org/10.5067/ASTER/ASTGTM.003) and the NASA Shuttle Radar Topography Mission Global 1 arc second V3 (SRTMGL1 v3; NASA/JPL, https://doi.org/10.5067/MEaSUREs/SRTM/SRTMGL1.003). These datasets are publicly available through the NASA Earthdata platform (https://earthdata.nasa.gov/). Data gaps in the ASTER GDEM were filled using SRTMGL1 v3 data.

Weirs, a prevalent type of low-head hydraulic structure in mountainous river systems, are engineering constructions typically under 5 meters in height, built for flow control, diversion, or water level elevation. According to a continental-scale survey by Belletti et al. [1], weirs account for approximately 30.5% of the 1.2 million documented river barriers across 36 European countries. Among records with height data, 91% of barriers are below 5 m, averaging about 2.77 m in height. To systematically characterize the distribution of weirs in the study region, we conducted a province-wide survey in Zhejiang Province, China (land area: 105,500 km²), based on the 2022 Zhejiang Water Domain Survey Database and high-resolution remote sensing imagery. We estimated a total of over 50,000 weirs across the province (Fig 1). Specifically, we identified 7,326 weirs in 557 hilly and mountainous rivers with a drainage area > 100 km², corresponding to an average density of 0.83 weirs per river kilometer.

Historically, due to a lack of systematic planning, many weir systems suffer from engineering defects, including excessively dense spacing, overly high structures, steep slopes, and the absence of fish passage facilities. These features directly exacerbate fluvial habitat fragmentation—the physical disruption of hydrological connectivity, sediment transport, and organism migration by dense artificial barriers. Studies indicate that the cumulative impact of such low-head barriers significantly surpasses that of fewer large dams and is a dominant cause of longitudinal connectivity loss, or habitat fragmentation, in river systems [1]. Fragmentation not only destroys the floodplain-deep channel structure but also fundamentally alters hydro-geomorphic processes and ecological functions by impeding sediment transport, disrupting flow regimes, and severing migration and gene-flow pathways. Consequently, it accelerates ecological homogenization of aquatic ecosystems [2,3] and drives juvenilization of biotic communities [4,5], collectively undermining riverine ecosystem integrity and resilience [2–7].

In practical engineering applications, two primary structural configurations are commonly employed on the downstream face of such low-head weirs: sloped-channel or stepped-pool designs.

- **Sloped-channel Configuration:** This design typically consists of a continuous, uniformly inclined chute. The flow accelerates gradually in an open-channel regime, with its energy primarily dissipated through friction along the slope.
- **Stepped-pool Design:** This design is characterized by a series of alternating steps and pools. Energy is dissipated progressively through drops and hydraulic jumps, creating alternating zones of turbulent and placid flow.

These two configurations differ notably in physical form and hydraulic behavior. Sloped channels generally have steeper slopes, higher velocities, and a relatively uniform flow regime. In contrast, stepped pools interrupt continuous flow with drops, creating varied velocity zones and turbulent structures, and offering resting areas in pool sections where flow is subdued. For sloped-channel configurations, fishway efficiency is primarily limited by hydraulic parameters such as flow velocity, water depth, and flow state, which must be tailored to the swimming capabilities of target species—a topic that has received considerable research attention [8–14]. However, research on stepped-pool configurations necessitates a focus on fish leaping biomechanics and weir arrangement. Quantitative predictive models indicate that maximum fish leap height and natural leaping behavior are governed by multifactorial interactions encompassing ethological patterns [15], locomotor capacity [2], and hydraulic parameters [16]. Kinematic analyses of leaping behavior have identified optimized hydrodynamic thresholds for the passage of specific species such as *Atlantic salmon* (*Salmo salar*) [17] and *Iberian barbel* (*Luciobarbus bocagei*) [18]. Furthermore, biomechanical assessments have quantified the leaping abilities across diverse fish taxa [19], and empirical studies have evaluated passage efficacy and population dynamics under varying hydraulic regimes [20]. Recent methodological advancements include the proposal of a generalized leaping framework for heterogeneous barrier systems [21] and the development of automated trajectory analysis for launch parameters [22].

Current standardization protocols, such as those from China [23], the United States [24], and Canada [25], typically mandate a maximum step height of 0.3 m for pool-type fishways. However, these guidelines and the underlying research are mostly derived from large anadromous fish species (e.g., Salmonidae, Acipenseriformes). Their applicability to small stream-dwelling cyprinids remains largely unvalidated, and comprehensive species-specific data for *A. fasciatus* are still scarce, which hinders the ecological restoration of fragmented mountainous rivers in China.

*A. fasciatus* (Cypriniformes, Barbinae) is a small-bodied freshwater fish of ecological and economic importance [26–28], primarily distributed in the mid- to upper reaches of mountainous streams within the Yangtze River, Pearl River, and southeastern coastal basins of China, with additional populations in the Nam Ou River system [26,29–31]. Although studies on related species such as *Acrossocheilus paradoxus* have provided useful references for engineering—including assessments of swimming performance [32–34] and general weir-passage capacity [35]—comprehensive empirical data specific to *A. fasciatus* remain scarce. Therefore, in this study, *A. fasciatus* was selected as a model species to investigate upstream migration behavior through controlled hydraulic experiments. Key parameters including passage success rate (PSR), migration strategies, takeoff position, maximum leaping height, and leaping distance were systematically quantified across different body lengths. These findings may help inform future related research and provide a potential biomechanical basis for assessing engineering measures aimed at restoring habitat connectivity for *A. fasciatus*.

This study aims to provide an empirical basis for understanding and potentially mitigating the impact of low-head weirs on *A. fasciatus*. Specifically, it seeks to systematically quantify the passage behavior and key biomechanical parameters of *A. fasciatus* across a range of body lengths as they negotiate stepped weirs under controlled flume conditions. The investigation focuses on: (1) the passage success rate (PSR) at consecutive weirs; (2) the proportional use of two distinct passage strategies (Pure Leaping vs. Leaping-Swimming) and their relationship with body length; and (3) kinematic parameters, including takeoff position, leap height, and leap distance. Based on existing studies on fish locomotion and weir passage behavior [15,16,32], we established the following hypotheses: (1) Passage success rate (PSR) varies significantly with body length; (2) Larger individuals will predominantly adopt the Pure Leaping Strategy (PLS), while smaller individuals will rely more on the Leaping-Swimming Strategy (LSS); (3) Key kinematic parameters (including leap distance and takeoff position) will exhibit significant positive correlations with body length, while the correlation between leap height and body length will be weaker.

## Materials and methods

### Weir distribution survey

To establish the macroscopic context of river fragmentation in Zhejiang Province, we conducted a weir distribution survey as background for this study. The survey comprised two complementary parts: (1) For the provincial total estimate, data were compiled and aggregated from the Zhejiang Water Domain Survey Database; (2) To obtain the linear density (weirs per river kilometer) for major rivers, we focused on hilly and mountainous rivers with drainage areas exceeding 100 km². Weirs within these river courses were identified and counted via interpretation of remote sensing imagery, primarily based on high-resolution satellite imagery available on platforms such as the ASTER Global Digital Elevation Model V3. Identification was based on the presence of permanent structural features that visibly impound water and create a discernible head difference across the channel. Total number and linear density were then calculated. This survey was descriptive and aimed to provide regional context; the data were not used in subsequent statistical analyses of fish behavior.

### Specimen collection and acclimatization

#### Sample collection.

Experimental specimens of *A. fasciatus* were collected from the Majinxi River basin in Kaihua County, located in the upper reaches of the Qiantang River. All collection procedures complied with animal ethics guidelines and were approved by the local administration (Majin Town Government, Kaihua County). To minimize harm and stress, fish were captured by experienced local fishermen using minnow traps or similar fish-friendly enclosures. Upon capture, individuals were immediately transferred to oxygenated transport tanks and promptly delivered to the laboratory for acclimatization.

#### Acclimatization and rearing conditions.

After arrival, fish were transferred to an indoor recirculating aquaculture system for a minimum acclimatization period of 14 days to ensure recovery and acclimation to laboratory conditions [8,19,21]. The system maintained water quality parameters (temperature, pH, dissolved oxygen, hardness) closely matching their natural habitat. Dechlorinated tap water (aerated for 48 hours) was used, with key parameters maintained as follows: water temperature at 22°C, dissolved oxygen ≥7.0 mg/L, and ammonia nitrogen <0.01 mg/L, under ambient indoor lighting. Fish were fed daily with a diet approximating their natural food sources. A stocking density of approximately 50 fish per cubic meter was maintained. Specimens were fasted for 48 hours prior to experimentation.

### Sample grouping and age estimation

For experimental observation, all specimens (*N* = 640) were divided into four major groups based on total length: < 9 cm (mean 8 cm), 9–11 cm (mean 10 cm), 11–13 cm (mean 12 cm), and > 13 cm (mean 15 cm), with 160 individuals per group. Each major group was further divided into two subgroups. The two replicate trials for each body length class were conducted using different batches of fish, with 80 fish per batch, thereby ensuring the statistical independence of all eight trial groups. Individual age was estimated based on a previously established body length–age relationship for *Acrossocheilus parallens* (*A. parallens*) [36,37]. According to this model, individuals with a total length <9 cm were estimated to be < 2 years old, those measuring 9–11 cm corresponded to 2–3 years, 11–13 cm to 3–5 years, and > 13 cm to > 5 years. Although recent taxonomic studies suggest *A. parallens* and *A. fasciatus* may be conspecific [38,39], more importantly, the two are highly similar in total length, external morphology, and key ecological traits such as adaptation to fast-flowing streams, algae grazing, and male egg-guarding behavior [28]. Given this high morphological and ecological convergence, we referenced the *A. parallens* growth model as a reasonable proxy for our study species.

### Experimental setup and hydraulic condition

#### Experimental setup.

**Fig 2:**
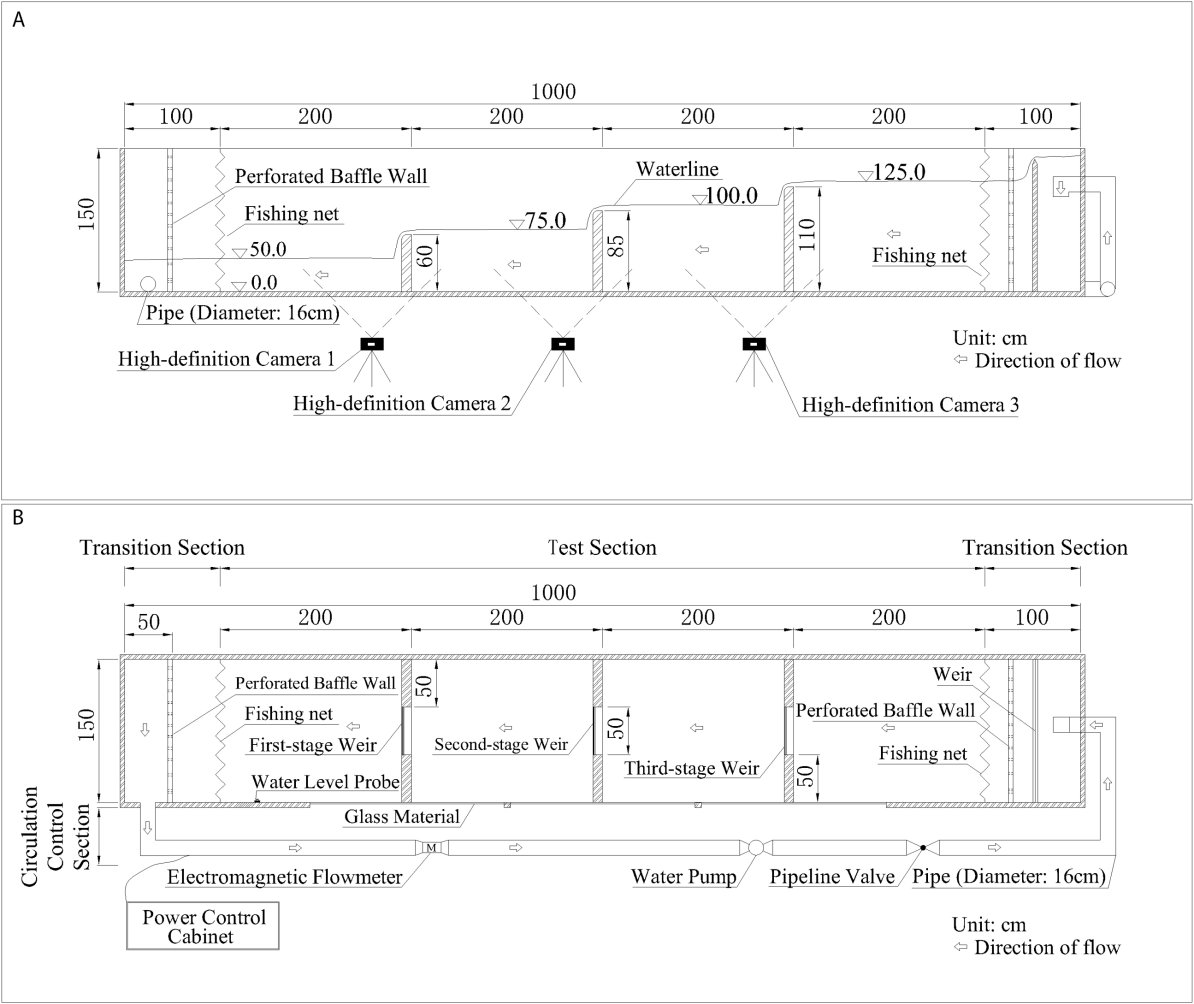
Configuration of the experimental setup (Unit: cm). **(a)** Plan view; **(b)** Section view.

We constructed a self-circulating flume system featuring a stepped-weir structure for the experiments (Fig 2, S1 File). The flume was 1000 cm long, 150 cm wide (weir crest width: 50 cm), and 150 cm high. It incorporated three vertically spaced low weirs with a 25 cm drop between successive stages. The system comprised three functional sections:

- **Transition Section:** Designed to smooth incoming flow and establish a stable hydraulic entry condition for the test section.
- **Test Section:** The core area for observing and recording fish upstream migration behavior (e.g., takeoff, leaping) and corresponding flow patterns.
- **Circulation Control Section:** Housing the pump, filter, and piping responsible for water circulation and regulation to maintain required hydraulic conditions.

Key apparatus included a pump, an electromagnetic flowmeter, pipeline valves, and a variable frequency drive for precise flow control. Throughout trials, water temperature was stabilized at 20 ± 0.5 °C and dissolved oxygen maintained at ≥ 7.0 mg/L. Fish behavior was recorded by three time-synchronized high-definition cameras (Sony FDR-AX30). A pre-calibrated scale on the flume’s inner wall provided spatial reference.

#### Hydraulic condition setup.

Target hydraulic conditions, simulating mountainous stream ecological flows [40], were set as: flow rate = 8.0 L/s; hydraulic head (water level difference) per weir = 25–26 cm; mean forebay velocity = 0.35 m/s; weir crest depth = 3–5 cm; and weir crest velocity = 0.65 m/s. Preliminary trials calibrated pump frequency and water volume to achieve stable target conditions. The system remained static for 48 hours before formal testing.

### Experimental procedure

For formal experiments, the calibrated flow conditions were re-established and allowed to stabilize for 30 minutes. A total of 640 fish were used, divided into four body-length groups: < 9 cm (mean ~8 cm), 9–11 cm (mean ~10 cm), 11–13 cm (mean ~12 cm), and > 13 cm (mean ~15 cm), with 160 individuals per group (age estimated as previously described). Testing proceeded sequentially from the largest to the smallest size group. For each trial, 80 fish from the same body length group were gently introduced into the downstream acclimation zone of the flume. After a 30-min acclimation period, their upstream movement and leaping behavior were recorded for 2 hours under stable hydraulic conditions, with no forced stimulation applied during the entire trial. After observation, fish were captured using soft nets and held temporarily. This was repeated for all batches within a size group, and the entire process was conducted twice (two replicates).

### Data acquisition and processing

#### Data monitoring protocol.

The experiment was conducted under constant hydraulic conditions. Data focused on leaping behaviors across body lengths and included: the total number of leaps observed, leaping trajectory (comprising takeoff position, leap height, landing position), from which leaping success rate and passage strategy were analyzed.

- **Data Acquisition:** Trials were recorded from orthogonal views using two synchronized high-speed cameras. A calibration scale affixed to the flume wall allowed for video calibration and scale conversion. Kinematic data were extracted via manual frame-by-frame video analysis.
- **Coordinate Definition:** The intersection of the downstream vertical weir face and the free water surface was set as the coordinate origin (0,0). A pre-calibrated steel tape was affixed along the flume wall to establish the coordinate system and provide spatial scaling. Fish movement trajectory was obtained by tracking the snout tip position.

### Behavioral parameter definitions

- **Passage Success Rate (PSR):** The percentage of tested fish that successfully passed over the weir.
- **Takeoff Position:** The location where the fish’s snout tip broke the water surface at leap initiation, expressed as the horizontal distance from this point to the coordinate origin.
- **Leap Distance:** The horizontal distance between the takeoff position and the landing position where the fish’;s snout tip re-entered the water, representing the forward propulsion achieved in a single leap.
- **Leap Height:** The maximum vertical elevation reached by the snout tip during a leap, expressed as the vertical distance from this point to the free water surface at the origin.
- **Passage Strategy Classification:** Two distinct passage strategies were defined based on high-speed video trajectory analysis: (1) Pure Leaping Strategy (PLS): Successful passage completed by a single continuous leap, where the fish’s body remained fully out of the water after takeoff, with no contact with the weir nappe, and landed directly on the weir crest. (2) Leaping-Swimming Strategy (LSS): Successful passage completed by an initial leap followed by continuous burst swimming within the weir nappe.
- **Effective Observation Period:** The formal observation duration was 2 hours. The total trial duration was 2.5 hours, with the initial 0.5 hours designated as a fish acclimation period (during which almost no upstream movement was observed). Leaping activity decreased markedly near the 2-hour mark, occurring only sporadically thereafter.

### Statistical analysis

To address the non-independence of data arising from testing unmarked fish in groups, all quantitative statistical analyses were performed at the level of independent trial groups (*N* = 8 total: 4 body length classes × 2 biological replicates per class). The observation from each trial group (e.g., PSR, median kinematic value, strategy proportion) was treated as a single, independent data point. All between-group comparisons and correlation analyses were based on these 8 data points. Regarding “upstream migration initiation,” due to the inability to distinguish individuals, we only provide descriptive notes and refrain from formal statistical inference. Pearson’s correlation coefficient (*r*) and coefficient of determination (*R*²) were used to quantify linear relationships. Linear regression was used to fit trends, and the significance of regression models was tested using ANOVA (*p* < 0.05). All statistical analyses were conducted using OriginPro 2021 (OriginLab Corporation, Northampton, MA, USA).

### Ethical statement and animal welfare

This study was approved by the Institutional Animal Care and Use Committee (IACUC) of Sichuan University (IACUC approval number: SCU44-2512-04) and all experiments were conducted under cooperative ethical authorization. All procedures involving fish were designed and conducted in accordance with the approved protocol, following principles of minimal intervention and welfare assurance. The experimental flume had smooth surfaces and protective nets to prevent injury. Handling was performed in water or moist conditions using soft nets, with air exposure limited to ≤ 20 s. No forced barriers, electrical, or chemical stimuli were used. Fish health was monitored daily, no fish mortality or significant injury occurred during the acclimation period and formal experiments. Post-experiment, all fish underwent a 48-hour recovery observation period. Prior to release at the original collection site, water conditions were gradually adjusted to match the natural habitat. This protocol was implemented throughout the study to ensure ethical standards and behavioral observation validity.

## Results

### Passage success rate across body length classes

**Fig 3:**
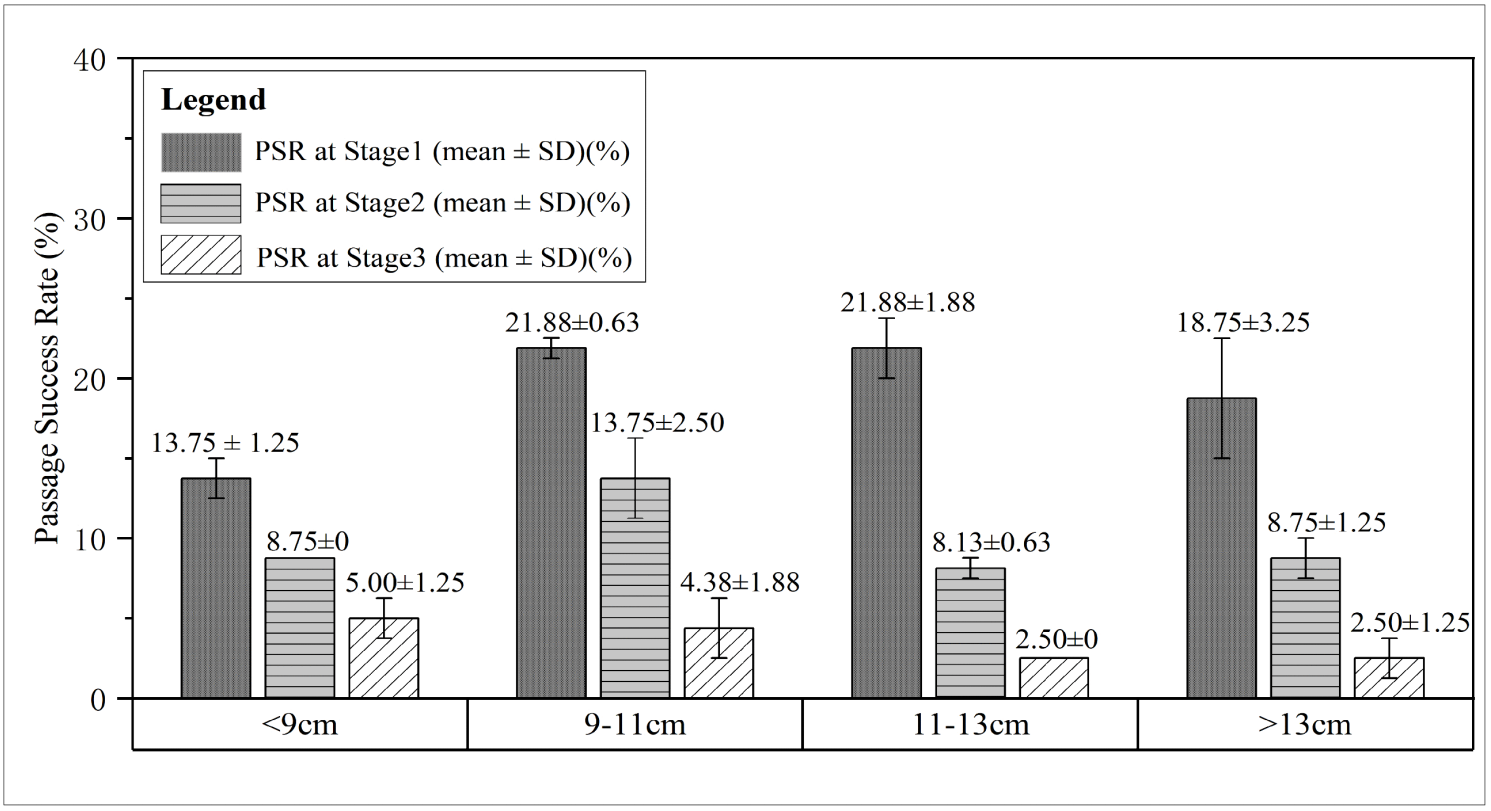
Passage success rate (PSR) of *A. fasciatus* at three successive weir stages across four body length classes. Bars represent the mean ± standard deviation (SD) of the two biological replicate trials within each body length class. All inferential statistical analyses in this study are based on the 8 independent trial group values (4 classes × 2 replicates).

The passage success rates (PSR) of *A. fasciatus* across different body length classes are summarized in Table 1 and shown in Fig 3. The data indicate an association between body length and passage performance, though not a simple monotonic relationship. Trial groups with mean body length 9–11 cm demonstrated the best cumulative passage performance (Total PSR), with a median of 40.0% (range: 35.0%–45.0%). This was followed by groups with mean body length 11–13 cm (median 32.5%, range: 30.0%–35.0%) and groups with mean body length >13 cm (median 30.0%, range: 23.75%–36.25%). Groups with mean body length <9 cm showed the lowest median cumulative success rate of 27.5% (range: 25.0%–30.0%). The “Overall Summary” row in Table 1 presents the full range (25.0%–45.0%) and overall median (33.13%) of Total PSR across all 8 independent trial groups, which form the sole basis for all inferential statistical analyses in this study.

**Table 1:** Passage success rate of *A. fasciatus* at successive weir stages.

Body Length (cm)	No. of Trials (*N*)	PSR at Stage 1(%) (Range, Median)	PSR at Stage 2(%) (Range, Median)	PSR at Stage 3(%) (Range, Median)	Total PSR (%) (Range, Median)
< 9	2	12.50-15.00(13.75)	8.75(8.75)	3.75-6.25(5.00)	25.00-30.00(27.50)
9-11	2	21.25-22.50(21.88)	11.25-16.25(13.75)	2.50-6.25(4.38)	35.00-45.00(40.00)
11-13	2	20.00-23.75(21.88)	7.50-8.75(8.13)	2.50(2.50)	30.00-35.00(32.50)
> 13	2	15.00-22.50(18.75)	7.50-10.00(8.75)	1.25-3.75(2.50)	23.75-36.25(30.00)
Overall Summary	8	12.50-23.75(19.69)	7.50-16.25(10.16)	1.25-6.25(3.75)	25.00-45.00(33.13)

Note: The table presents the passage success rate (PSR) of *A. fasciatus* through successive weir stages, grouped by body length class. The experimental design included four body length groups (< 9 cm, 9–11 cm, 11–13 cm, > 13 cm), with 2 trial replicates per group, resulting in a total of 8 independent trial groups. The PSR for each stage and the total are presented as percentages in the format of “range (median)” derived from the trial replicates per group. The “Overall Summary” row provides the overall range (minimum – maximum) and the median of all 8 independent trial observations for each metric, summarizing the central tendency and full variation across the entire experiment.

At the stage-specific level, all body length classes exhibited a consistent and sharp decline in PSR with increasing weir stage. The first weir (Stage 1) had the highest passage rates (median range across groups: 15.00%–22.50%), which dropped markedly at the second stage (Stage 2; median range: 7.50%–16.25%), and were lowest at the third stage (Stage3; median range: 1.25%–6.25%). It is noteworthy that the 9–11 cm class maintained relatively higher median passage rates at both Stage 2 and Stage 3 compared to other groups, which likely contributed significantly to its superior cumulative performance.

### Analysis of upstream migration strategies

**Fig 4:**
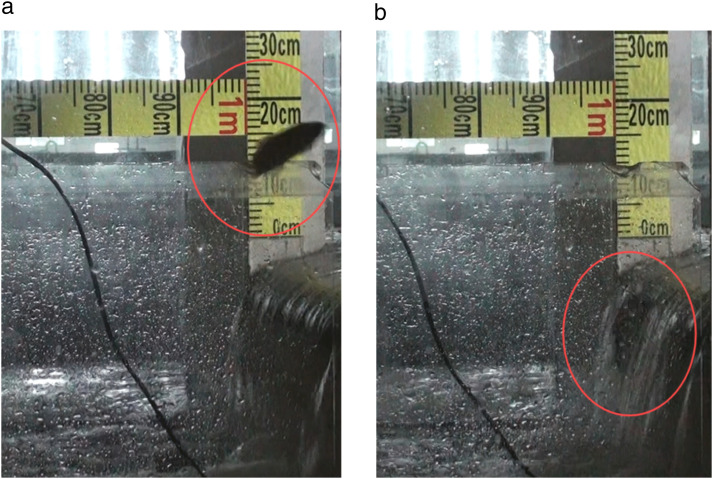
Photographs of *A. fasciatus* during upstream passage attempts. **(a)** PLS; **(b)** LSS. Leaping individuals are marked with red circles.

Kinematic analysis of movement trajectories identified two distinct passage strategies employed by *A. fasciatus*: a Pure Leaping Strategy and a Leaping-Swimming Strategy (Fig 4).

**Strategy description and body length dependence:**

- **Pure Leaping Strategy (PLS):** Individuals using the PLS initiated leaps from a point outside the nappe and landed directly on the weir crest, executing high-arc trajectories (Fig 4a).
- **Leaping-Swimming Strategy (LSS):** Individuals using the LSS typically executed trajectories closer to the weir face, involving contact with and swimming within the nappe to ascend (Fig 4b).

Movement trajectory analysis at the trial-group level revealed a clear body size-dependence population pattern: trial groups with larger mean body length had a significantly higher proportion of successful passages using the long-range, arcing leaps characteristic of PLS, whereas groups with smaller mean body length showed a higher proportion of passages using the near-weir trajectories associated with LSS (Fig 4).

**Quantification of strategy usage proportion:**

**Fig 5:**
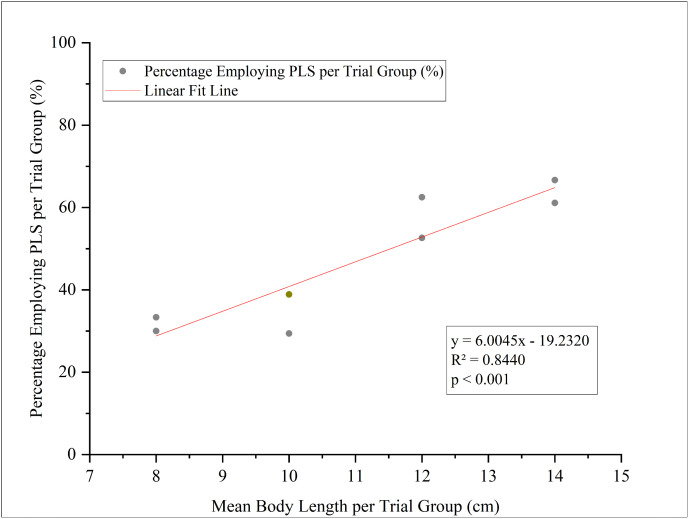
Relationship between mean body length per trial group and the proportion of PLS/LSS usage. Each data point represents one of the 8 independent trial groups. All linear regression analyses are based on these 8 independent data points..

The proportion of PLS usage increased strongly with body length at the trial-group level (Fig 5, Table 2). Linear regression analysis at this level indicated that the mean body length of a trial group explained a substantial proportion of the variance in PLS usage (*R*² = 0.844, *p* < 0.001; Fig 5). Specifically, the proportion of fish employing PLS at the first weir increased from a median of 31.82% in the smallest size class (< 9 cm) to 63.33% in the largest size class (> 13 cm) (Table 2). Conversely, the use of LSS decreased correspondingly. Across all independent trials, PLS and LSS accounted for 46.65% and 53.35% of successful passage events, respectively (Table 2, Overall Summary). This quantified shift confirms that body length is a key determinant of passage strategy in *A. fasciatus*.

**Table 2:** Proportion of passage strategy usage of *A. fasciatus* at the first weir, by body length class..

Body length class (cm)	No. of independent trials	Percentage employing PLS (%, Range, Median)	Percentage employing LSS (%, Range, Median)
< 9	2	30.00-33.33(31.82)	66.67-70.00(68.18)
9-11	2	29.41-38.89(34.29)	61.11-70.59(65.71)
11-13	2	52.63-62.50(57.14)	37.50-47.37(42.86)
> 13	2	61.11-66.67(63.33)	33.33-38.89(36.67)
Overall Summary	8	29.41-66.67 (46.65)	33.33-70.59 (53.35)

Note: Data represent the percentage of fish employing each strategy during successful passage of the first weir, presented as the range (minimum – maximum) and median (in parentheses) observed across the 2 independent trial replicates per body length class. The “Overall Summary” row summarizes the full data spread (*N* = 8 independent trial groups) to illustrate the population-level strategy shift with body length, without implying a statistical average of group summaries.

### Analysis of parameters associated with upstream migration

#### Key kinematic parameters analysis.

**Fig 6:**
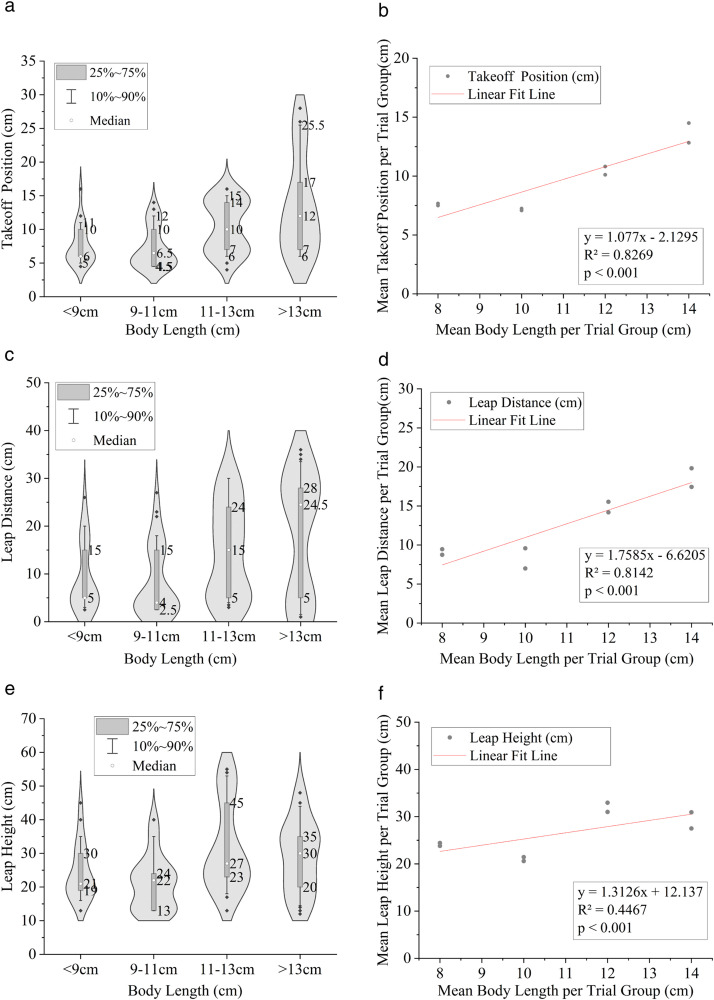
Behavioral parameter analysis of upstream migration in *A. fasciatus* of different body lengths. **(a) (b)** Relationship between body length and takeoff position; **(c) (d)** Relationship between body length and leap distance; **(e) (f)** Relationship between body length and leap height. Each data point represents one of the 8 independent trial groups, showing the mean body length and median kinematic value for that group. All linear regression analyses (trend lines) are based on these 8 independent data points, with *R*² and *p* values reported in the text.

Key kinematic parameters—takeoff position, leap distance, and leap height—showed systematic variation with the body length of *A. fasciatus* at the trial-group level (Fig 6).

- Takeoff Position: A strong positive correlation was observed between the mean body length of a trial group and its mean takeoff position (*R*² = 0.83, *p* < 0.001; Fig 6b). At the trial-group level, median takeoff position increased significantly with mean body length. The interquartile range (box span) of takeoff position also increased with body length, indicating greater variability among trial groups with larger mean body length.
- Leap Distance: The mean leap distance per trial group exhibited an extremely strong, positive dependence on mean body length (*R*² = 0.96, *p* < 0.001; Fig 6d). This demonstrates that trial groups with larger mean body length possess a substantially greater capacity for horizontal propulsion, which is critical for the successful execution of the Pure Leaping Strategy.
- Leap Height: In contrast, the relationship between mean body length and mean leap height was weaker (*R*² = 0.45, *p* < 0.001; Fig 6f). While a positive trend existed, leap height showed considerable variation across trial groups, indicating that it is not a simple, deterministic function of body length in this species under the tested conditions.

### Swimming phase in the leaping-swimming strategy

For individuals employing the Leaping-Swimming Strategy (LSS), the swimming phase following the initial leap was crucial for completing passage. Analysis of the swimming (sprint) distance covered after the leap (Table 3) revealed that trial groups with larger mean body length exhibited greater median burst swimming distances. Specifically, the > 13 cm body length class achieved the highest average swimming sprint distance (7.36 cm) and the maximum recorded distance (13 cm) across trials. The performance in this swimming phase effectively complemented the preceding leap, collectively contributing to the overall success of the LSS.

**Table 3:** Statistics of swimming (Sprint) distance within the leaping-swimming strategy for *A. fasciatus* of different body lengths.

Trial Group	< 9 cm (Average / Maximum)	9-11 cm (Average / Maximum)	11-13 cm (Average / Maximum)	> 13 cm (Average / Maximum)	Total (Average / Maximum)
Group 1	5.29 / 9	7.00 / 12	5.78 / 12	7.50 / 11	6.32 / 12
Group 2	6.13 / 10	4.90 / 10	5.17 / 8	7.29 / 13	5.81 / 13
Total	5.73 / 10	6.00 / 12	5.53 / 12	7.36 / 13	6.06 / 13

Note: All values are distances in cm. The transition from leaping to swimming phase for calculation was defined as the point when the fish enters the nappe (water tongue) or initiates vigorous tail-beating. These are descriptive statistics of individual-level kinematic data. No inferential statistics were performed on these values, as all quantitative analyses were conducted at the trial-group level.

## Discussion

### Synthesis of key findings and mechanistic implications

This study provides quantitative evidence, under specific laboratory conditions (a single hydraulic and structural configuration), that the upstream passage capacity of *A. fasciatus* over low-head weirs is primarily governed by body length, which in turn dictates the choice of migration strategy and key kinematic performance. Analysis at the independent trial-group level confirmed baseline passage capability among the fish under the tested conditions, with the passage success rate at the first weir ranging between 12.50% and 23.75% (median 19.69%) across all 8 independent trial groups. However, the success rate decayed extremely rapidly with increasing weir stage, dropping to only 1.25%–6.25% at the third weir, underscoring the severe cumulative fatigue effect induced by sequential barriers. The analysis also confirmed the hypothesized ontogenetic shift in strategy usage pattern: trial groups with mean body length >13 cm showed a significantly higher proportion of PLS usage, relying on powerful bursts to clear the weir in a single jump, whereas groups with mean body length ≤13 cm showed a higher proportion of LSS usage, combining a shorter jump with subsequent swimming through the nappe (Figs 4–6). This strategic divergence is underpinned by distinct biomechanical capabilities. We found an extremely strong positive trend between body length and leap distance at the trial-group level (*R*² = 0.96), enabling trial groups with larger mean body length to initiate leaps from farther downstream and achieve the greater horizontal propulsion required for PLS. In contrast, the association between body length and leap height was weaker (*R*² = 0.45), indicating that vertical clearance was not a primary limiting factor, particularly for PLS (Fig 6f). This weaker relationship challenges the generalized leap height estimation model based solely on body length (LH = (9L)²/2g) proposed by Lauritzen et al. [2,16], suggesting its limited applicability to small-bodied cyprinids such as *A. fasciatus*.

### Comparative analysis with other small-bodied fish species

The behavioral strategies and performance thresholds observed in *A. fasciatus* align with and extend findings for other small-bodied cyprinids. For instance, the *Iberian barbel (Luciobarbus bocagei)*, a species of similar ecology, also employs a combined leap-swim tactic at broad-crested weirs, with success being highly contingent on local hydraulics [18]. Our conclusion that leap distance, not height, is critical for PLS success echoes research on *Acrossocheilus paradoxus*, which identified sufficient horizontal thrust as key for obstacle negotiation [35]. However, a key distinction emerges in the absolute performance metrics. The median leap heights for *A. fasciatus* (28.5–40 cm in this study) are substantially lower than those reported for *salmonids* [17] but are comparable to or exceed those of other small stream fish like some native cyprinids in the Great Plains [19]. This underscores the inadequacy of passage criteria developed for powerful leapers like salmonids when applied to small-bodied species. Furthermore, the strong body-length dependency of strategy usage pattern in *A. fasciatus* offers a more nuanced framework than studies proposing a single optimal strategy for a species [20], highlighting the necessity for size-structured considerations in fishway design.

### Limitations of flume experiments and field applicability

Several limitations of this study should be noted when interpreting the results, and the derived design implications must be interpreted strictly within the constraints of our methodological design. While controlled flume experiments enable precise variable isolation and biomechanical quantification, they inevitably simplify the complexity of natural river systems. The core limitations are as follows:

- Statistical and analytical constraints: The group-based testing design without individual fish marking precluded individual-level behavioral analysis. To avoid data non-independence and pseudo-replication, all statistical analyses were performed at the level of 8 independent trial groups, which limits the granularity of individual behavioral inference. In addition, the age of experimental fish was estimated using a growth model from a closely related *Acrossocheilus species*, with no direct validation via otolith analysis.
- Limited hydraulic and structural generalizability: Only a single fixed configuration (25 cm weir drop, 45° sloped crest, constant flow rate) was tested. The stable, uniform laboratory flow differs from the turbulent, heterogeneous flow fields at natural weirs, and the fixed unit discharge (≈ 0.016 m²/s) does not cover the full range of flow regimes fish encounter in the wild. This restricts the universal applicability of our results to other hydraulic conditions or weir geometries.
- Mismatch with natural biological context: Experiments were conducted on rested, non-spawning individuals under optimal laboratory conditions, which do not account for key factors affecting wild fish performance, including seasonal spawning motivation, fatigue accumulation, predation risk, water temperature fluctuations, and interspecific interactions or shoaling behavior in natural habitats.

Accordingly, the observational thresholds presented in this study are preliminary, conservative benchmarks derived from a specific laboratory setting, rather than universally applicable engineering standards. For future research, we recommend using Passive Integrated Transponder (PIT) tags or computer vision-based individual tracking to obtain precise individual behavioral data. Combined with multivariate methods such as Principal Component Analysis (PCA) [41,42], these data can systematically disentangle the complex relationships between fish passage strategies, kinematic performance, and hydrodynamic factors. Further multi-condition comparative tests and field validation under natural hydraulic conditions are essential to calibrate our findings and improve their engineering applicability.

### Design considerations for low-head weirs based on the behavior and protection strategies of *A. fasciatus*

**Fig 7:**
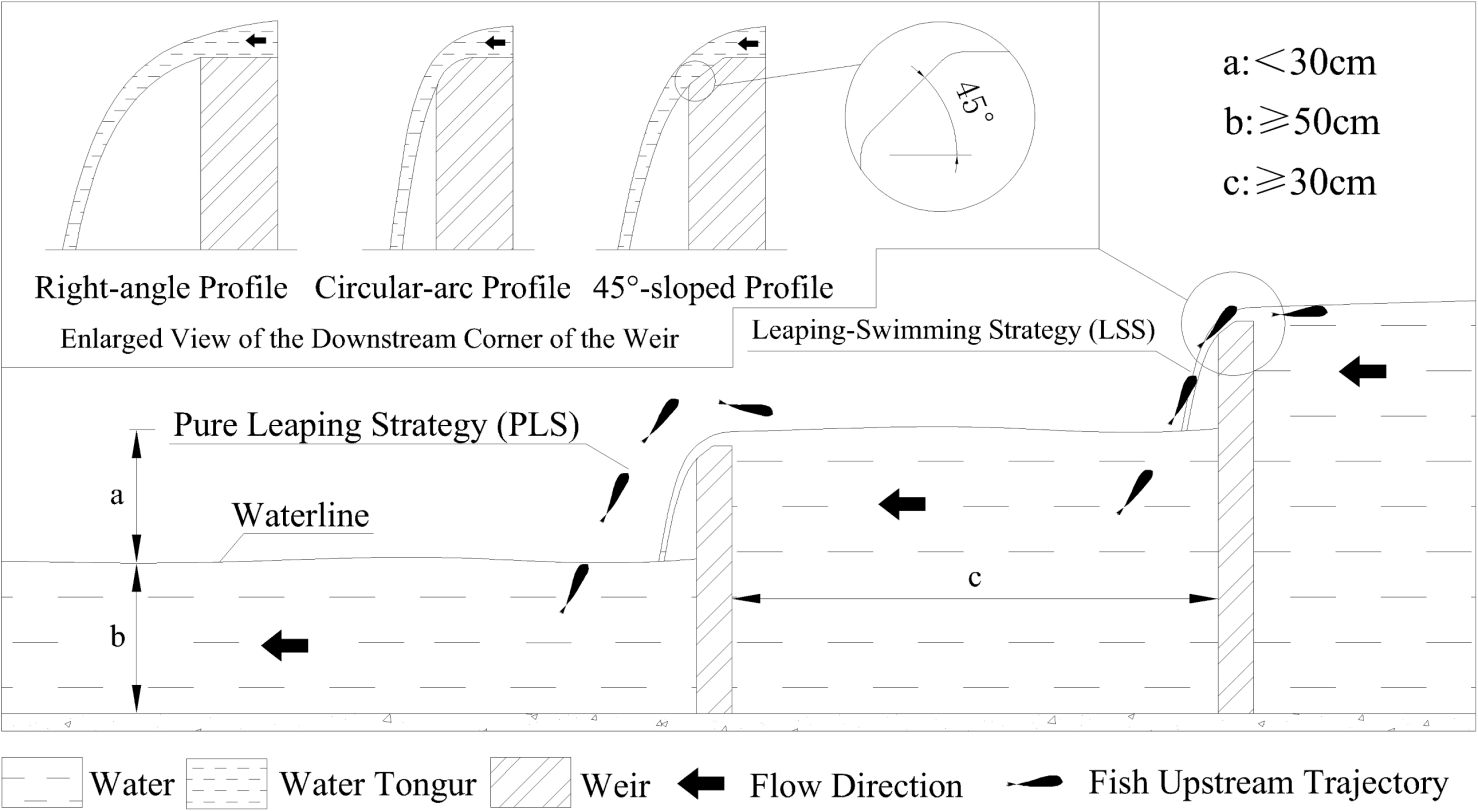
Schematic of weir crest profile and pool layout. **(a)** hydraulic head drop; **(b)** pool depth; **(c)** inter-weir pool length).

Based on the behavioral and kinematic observations from this single laboratory configuration, we present the following trends that may have potential implications for future eco-engineering research, as illustrated in Fig 7. These are not prescriptive design recommendations, but rather observational data that require validation through comparative experiments.

- Weir Height (Hydraulic Head Drop): The precipitous drop in cumulative PSR underscores the severe impact of cumulative fatigue. Combined with the observed median leap heights, this finding supports the existing literature [35] which suggests that, for *A. fasciatus*, a weir drop of 25–30 cm—under the tested conditions—enabled a degree of successful passage. For greater total elevations, a cascade of low-head drops may be more conducive to passage than a single high barrier, based on the observed severe cumulative fatigue effect.
- Weir Crest Profile: Strategy analysis revealed that over 60% of fish, particularly those ≤13 cm in body length, relied on the Leaping-Swimming Strategy (LSS), the success of which depends on their ability to adhere to and swim within the weir nappe. Based on observed fish behavior and analysis in conjunction with hydraulic principles, it is theorized that an inclined or rounded weir crest may generate a nappe with a gentler trajectory and a greater water thickness, thereby potentially providing a more stable zone for fish to execute the LSS [18]. However, it should be noted that this study utilized only a fixed 45° inclined weir crest as the test condition, with no comparative testing conducted against other weir crest geometries (such as a vertical sharp-crested weir or a flat-top weir). Consequently, the available data cannot support any conclusions regarding the relative performance of different weir crest profiles. The observation that smaller individuals (≤13 cm) predominantly used the LSS under this fixed condition does not imply that the 45° inclined crest is superior to other designs. Any inference that an inclined or rounded crest might be more favorable for LSS passage remains theoretical speculation at present and requires rigorous validation through comparative experiments. In summary, although the widespread use of the LSS was observed on the ≈ 45° sloped weir crest in this study, this does not constitute a comparative experiment of different geometries. The potential advantage of a sloped or arched weir crest remains a hypothesis derived from hydraulic theory and observations of strategy dependence, awaiting future experimental confirmation.
- Downstream Pool Dimensions: Following the principle that effective propulsion requires a sufficient plunge pool depth [16], and given a common adult length of ~15 cm, under the tested conditions, a downstream pool depth >30 cm was sufficient to accommodate the observed leaping kinematics. To accommodate the full leaping sequence for the largest individuals and considering the maximum observed leap distance (28 cm), an inter-weir pool length >50 cm was sufficient to accommodate the maximum observed leap distance.

### Applicability and caveats

The observations above are derived specificallyfrom the observed passage behavior and kinematic limits of *A. fasciatus* under the hydraulic conditions tested. They are not intended as prescriptive design standards. In particular, the suggestion regarding weir crest profile is a theoretically based inference rather than a conclusion drawn from comparative experimental evidence. For other species or different hydraulic regimes, these parameters may not be optimal. The primary value of this study lies in demonstrating a methodology and providing a species-specific dataset; applying these findings to engineering practice necessitates further site-specific assessment that integrates local hydraulics, target fish community, and cumulative barrier effects.

## Conclusions

This study fills a critical data gap in fish passage research by providing species-specific behavioral and biomechanical data for *A. fasciatus*, a representative small-bodied stream cyprinid that has been largely overlooked in existing design guidelines. Our findings highlight the importance of incorporating body length-dependent behavioral strategies into the eco-friendly design of low-head weirs, providing a preliminary quantitative basis for the ecological restoration of fragmented mountainous rivers. Three primary conclusions can be drawn.

First, *A. fasciatus* exhibits a clear body length-dependent shift in passage strategy usage patterns. Trial groups with mean body length >13 cm showed a higher proportion of PLS usage, while groups with mean body length ≤13 cm showed a higher proportion of LSS usage. This strategic shift underlies the differential passage performance among size groups.

Second, kinematic parameters show divergent correlations with body length. Mean leap distance per trial group is extremely strongly positively correlated with mean body length per trial group (*R*² = 0.96) and determines the success of PLS, whereas leap height is only weakly correlated (*R*² = 0.45), indicating that vertical clearance is not the primary limiting factor.

Third, under the specific laboratory conditions tested, we observed that a single weir drop of 25–30 cm, a downstream pool depth >30 cm, and an inter-weir pool length >50 cm were sufficient to allow partial passage of *A. fasciatus*. These observational values are limited to the tested hydraulic and structural configuration, and their generalizability to engineering practice requires validation through multi-condition experiments and field monitoring.

This study provides species-specific data to support the design of fish-friendly low-head weirs and highlights the importance of body length-specific behavioral strategies in fish passage assessment. All three hypotheses proposed in this study were supported at the trial-group level: (1) PSR of *A. fasciatus* varied significantly with mean body length per trial group; (2) Trial groups with larger mean body length showed a higher proportion of PLS usage, while groups with smaller mean body length showed a higher proportion of LSS usage; (3) Mean leap distance and median takeoff position were significantly positively correlated with mean body length per trial group, while the correlation between mean leap height and mean body length was weaker.

## Supporting information

S1 File Detailed introduction of experimental apparatus and research methods. (DOCX)
