# Supplementary material for: Upstream migration capacity of Acrossocheilus fasciatus: Behavioral strategies in response to hydraulic conditions and implications for low-head weir design
Source: PLoS One. 2026 May 29;21(5):e0350692. doi: 10.1371/journal.pone.0350692 (PMC13221079; doi:10.1371/journal.pone.0350692)
Supplement: S1 File — (DOCX) [file pone.0350692.s001.docx]

**Experimental Apparatus and Method**

This study employs physical model testing with a 1:1 scale model. The following sections describe the model design, experimental apparatus, rearing conditions, and testing methodology.

**1.Model Design**

The experimental system utilizes a self-circulating flume featuring a stepped weir structure, comprising three functional sections: a transition section, a test section, and a circulation control section. Transition Section: Primarily used to smooth the incoming flow and adjust the flow regime, providing stable hydraulic entry conditions for the test section.Test Section: The core experimental area for observing and recording the upstream migration behavior of fish (e.g., take-off, leaping) and the corresponding flow patterns.Circulation Control Section: Houses the pump, filter, and piping, responsible for water circulation, control, and regulation to maintain and alter the hydraulic conditions required for the experiments.The flume has a total length of 1000 cm, a width of 150 cm (test section weir crest width: 50 cm), and a height of 150 cm, with three vertically spaced low weirs featuring a 25 cm vertical drop between successive stages (fig.1, fig.2). During experiments, three time-synchronized high-definition cameras were used to record fish behavior. A pre-calibrated measurement scale was marked along the flume's inner wall for spatial reference. In post-processing, video frames were extracted for systematic analysis of leap frequency, trajectory mapping, and quantitative assessment of leaping behavior.

(a)

(b)

**Fig. 1 Configuration of the experimental setup (Unit: cm): (a) Plan view; (b) Section view.**


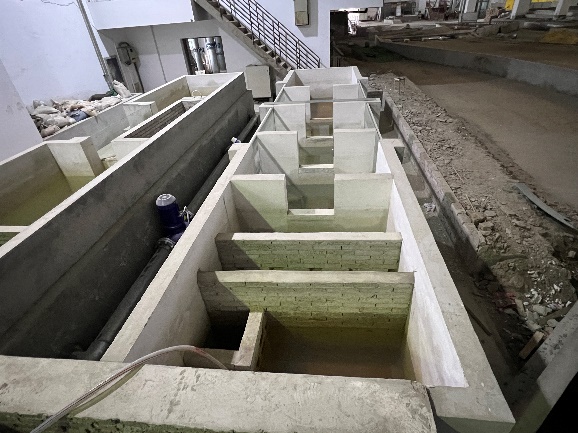

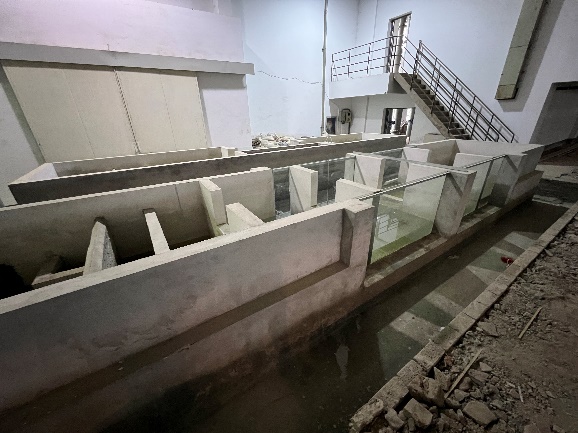


**Observation window**

**Removable weir plate**

**Perforated Baffle**

**Power Control Cabinet**

**Fig. 2 Photo of the experimental setup.**

**2.Experimental Apparatus**

**Experimental Apparatus:** Power Control Cabinet, Water Level Probe, Water Pump, Electromagnetic Flowmeter, Pipeline Valve, HD Camera, Pump Variable Frequency Drive Controller(Fig.3).


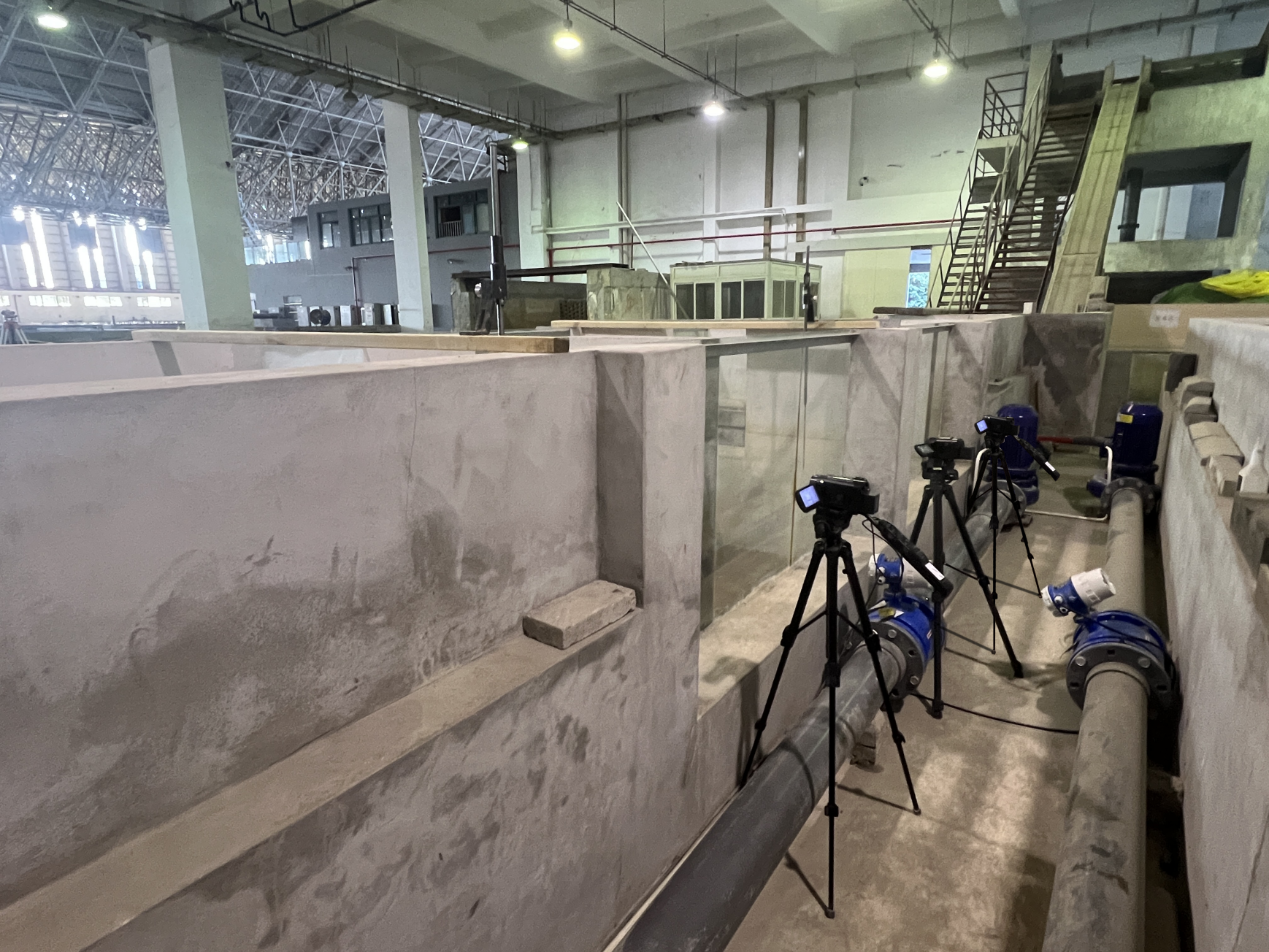


**HD Camera**

**Electromagnetic**

**Flowmeter**

**Pipeline Valve**

**Water Level Probe**

**Water Pump**

1. Experimental Apparatus Layout


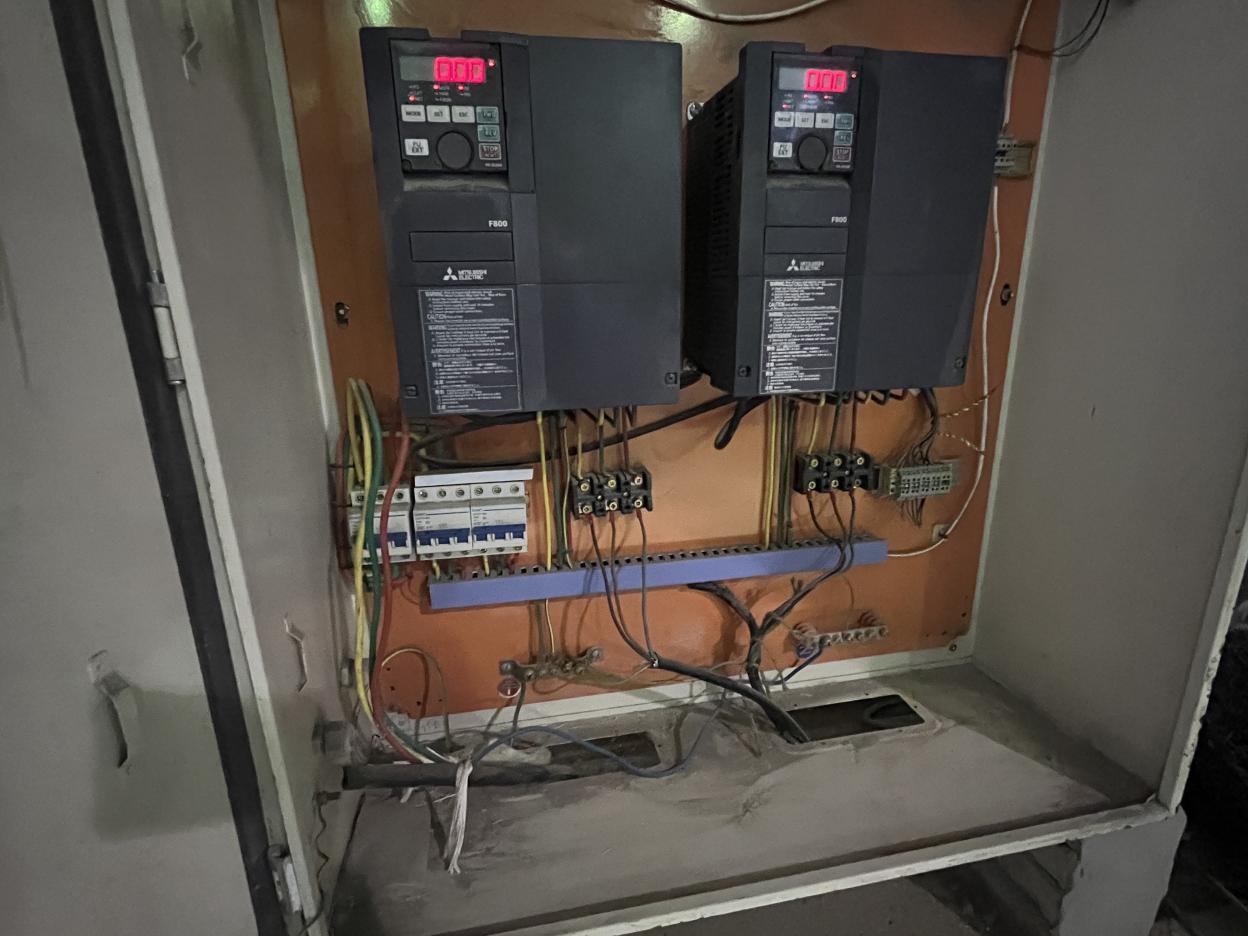

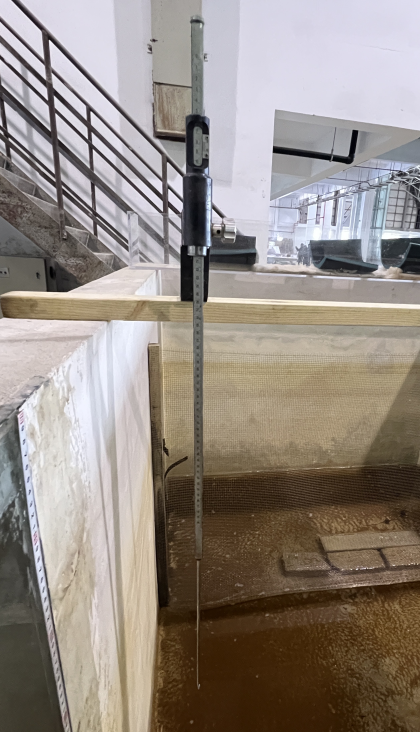


**Air Circuit Breaker (ACB)**

**Pump Variable Frequency Drive Controller.**

(b) Power Control Cabinet (c) Water Level Probe


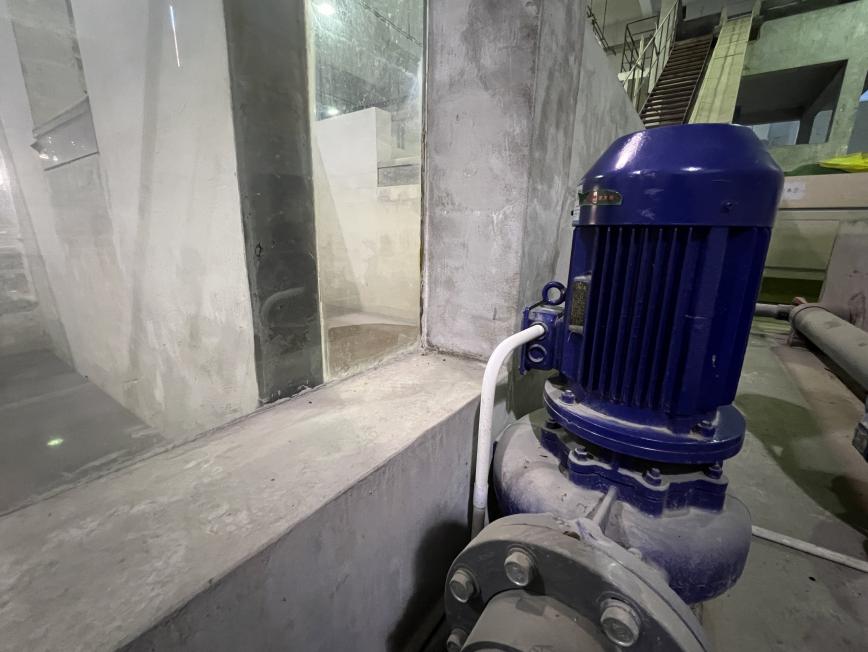

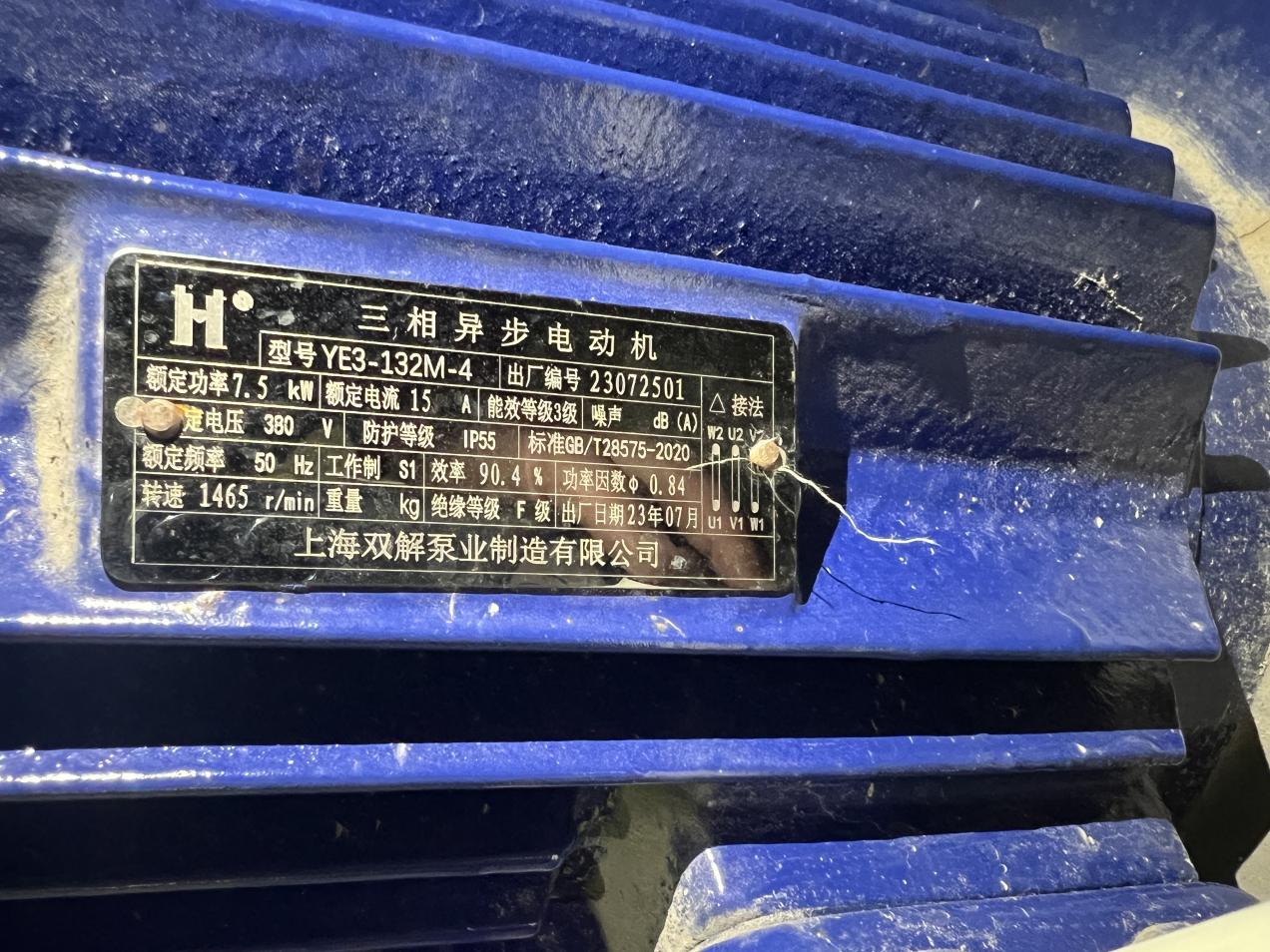


(d) Water Pump (A centrifugal pump powered by a YE3-132M-4 motor (rated power: 7.5 kW, voltage: 380 V, frequency: 50 Hz), with an inlet/outlet diameter of 150 mm, manufactured by Shanghai Shuangjie Pump Manufacturing Co., Ltd.)


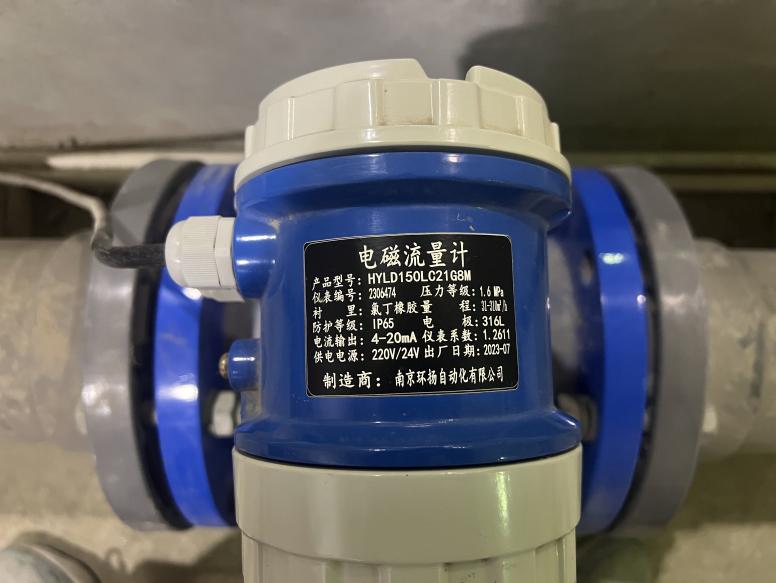

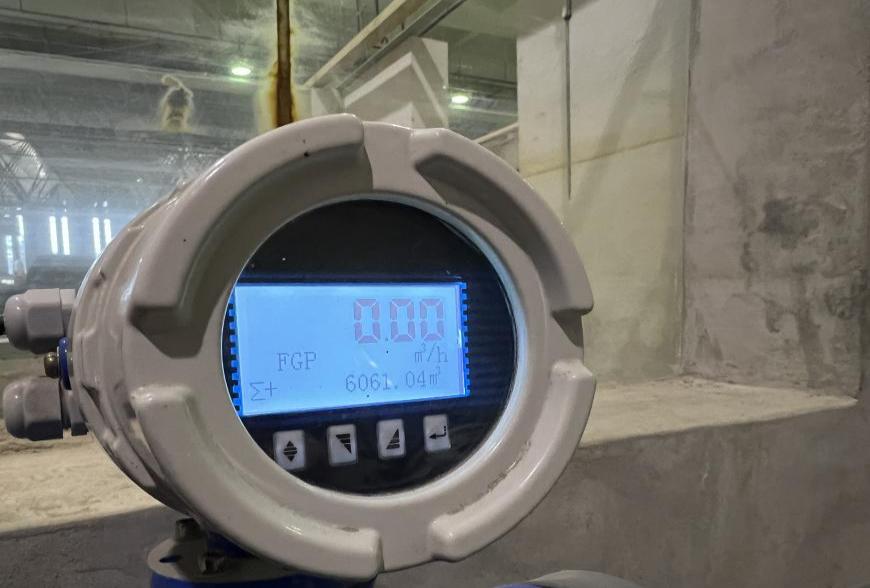


(e) Electromagnetic Flowmeter (Model HYLD150LC3216HM, Nominal Diameter (DN): 150, manufactured by Nanjing Huanhai Automation Co., Ltd. )


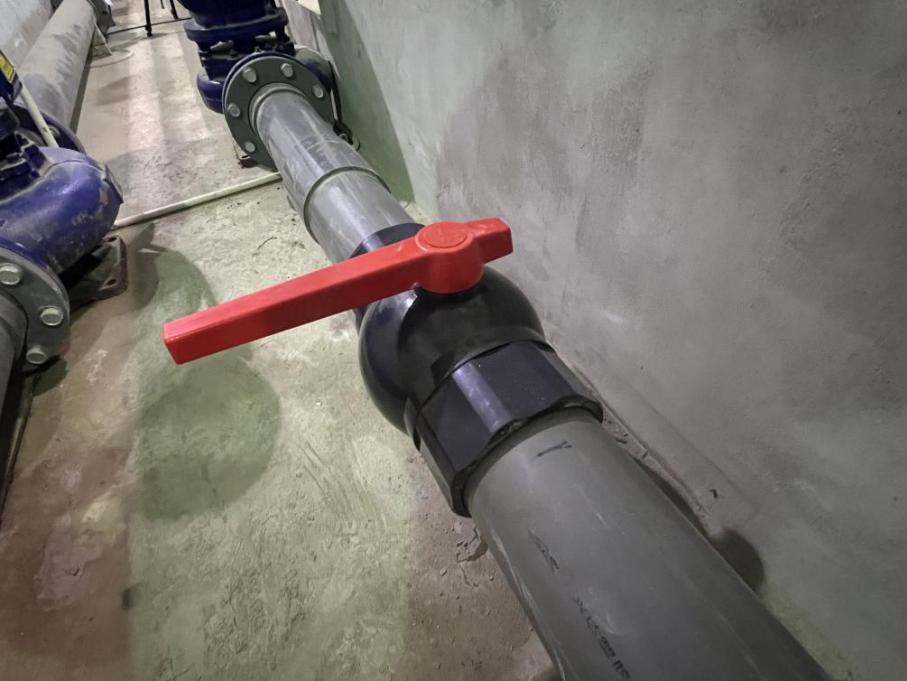

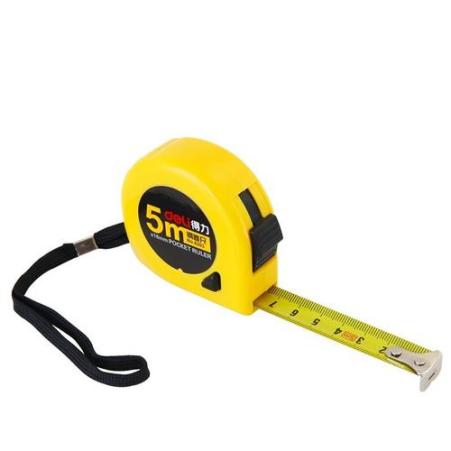


(f) Pipeline Valve (Pipe ID: 160 mm) (g) Steel Tape Measure(Deli-brand 5-meter , with a tape width of 1 cm and a finest graduation of 1 mm.)​


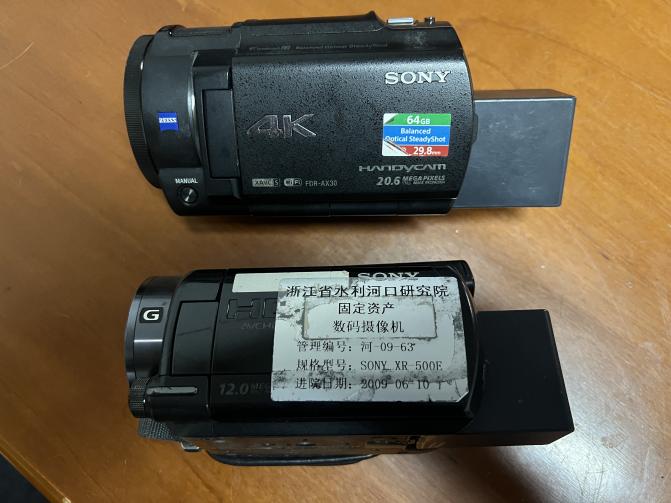


(h) HD Camera (Three time-synchronized cameras: one Sony FDR-AX30 and two Sony HDR-XR500E. Among them, the Sony FDR-AX30 features a maximum video recording resolution of 3840×2160 (4K UHD), with its HD specification being 1920×1080 pixels, covering frame rates of 50p/60p (at a maximum bit rate of 50 Mbps), 50i/60i, and 25p/30p; the Sony HDR-XR500E has a maximum video recording resolution of 1920×1080 (Full HD, without 4K shooting capability), with its HD specification being 1920×1080 pixels, supporting frame rates of 50i/60i (at a maximum bit rate of 24 Mbps) and 25p/30p.)


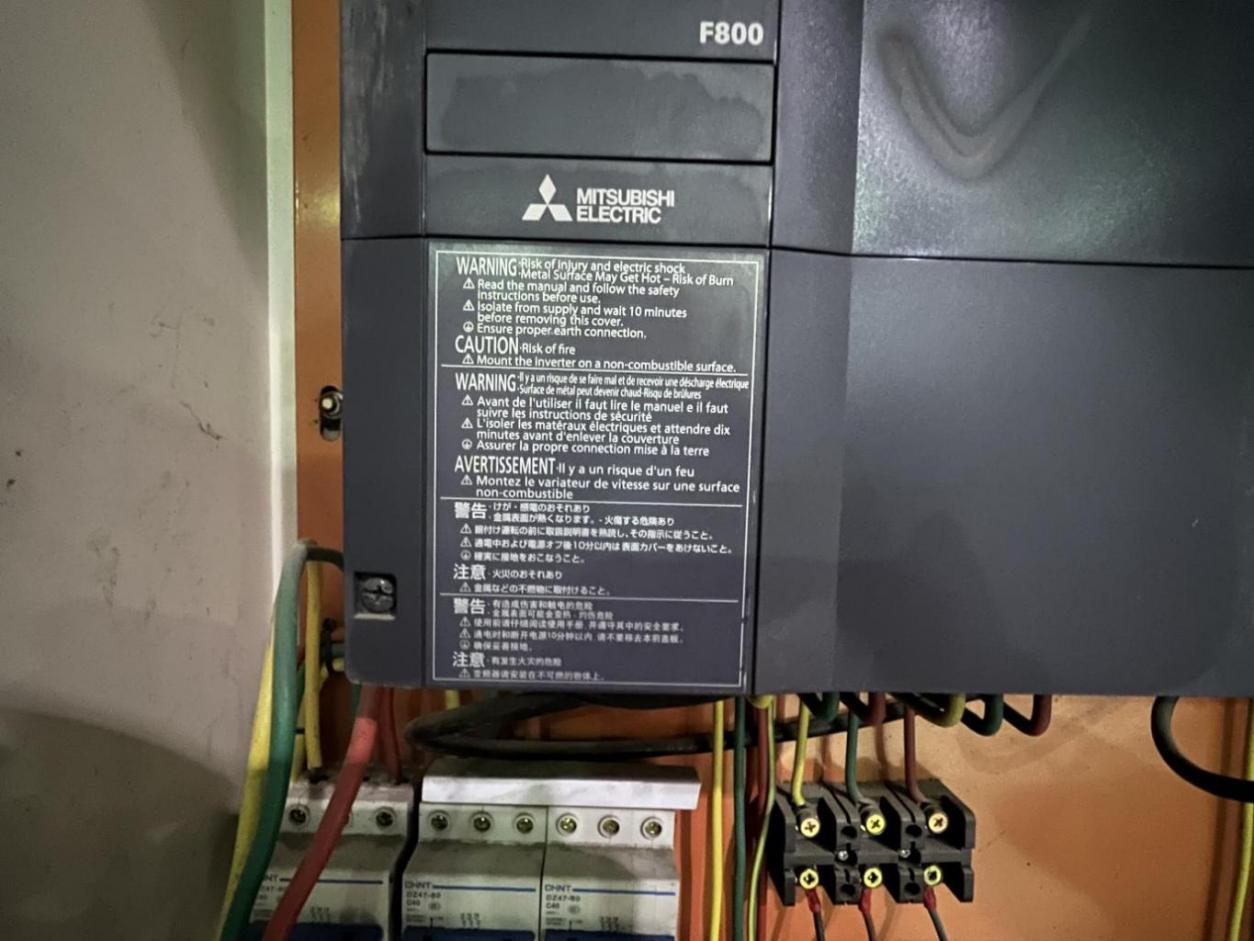


(i) Pump Variable Frequency Drive Controller (Variable Frequency Drive, Model F800, manufactured by Mitsubishi Electric Corporation)

**Fig. 3 Experimental apparatus.**

**3.Experimental Methods and Procedure**

**Step 1: Preliminary Trial**

- Close the valve on the recirculation pipeline. Add water to the uppermost chamber of the model until all chambers overflow. Stop adding water when the water levels in the two most downstream chambers become equal.
- Turn on, in sequence, the valve on the recirculation pipeline, the power supply for the pump variable-frequency speed controller, the pump power supply, and the electromagnetic flowmeter power supply.
- Adjust the frequency on the pump variable-frequency speed controller to change the pump power (flow rate) until the electromagnetic flowmeter displays a pipeline flow rate of 8 L/s.
- Slowly add water to the most downstream chamber of the model while simultaneously adjusting the frequency of the pump variable-frequency speed controller to change the pipeline flow rate. The goal is to simultaneously satisfy two conditions: a 25 cm water level difference (observed via water level probes) between the most downstream chamber and the weir crest, and an electromagnetic flowmeter reading of 8 L/s. It should be noted that after the pump is started, the water level in all chambers except the last one will rise, while the level in the final chamber will drop. Furthermore, due to the increased head difference between the first and last chambers, the flow rate displayed by the electromagnetic flowmeter will be less than 8 L/s. As water is added to the final chamber, the water level difference from the weir crest gradually approaches 25 cm. However, because the water depth over the weir crest increases by 3-5 cm, the final pipeline flow rate will be slightly less than 8 L/s. It is therefore necessary to slightly increase the frequency of the pump variable-frequency speed controller to raise the pump power and flow rate accordingly.
- Upon completion, record the readings from the electromagnetic flowmeter and the pump variable-frequency speed controller. Then, turn off the electromagnetic flowmeter, the pump power supply, and the pump variable-frequency speed controller power supply in sequence. Leave the recirculation pipeline valve in its current position and let the water in the model remain static for 48 hours.

**Step 2: Formal Experiment**

- Turn on, in sequence, the power supply for the pump variable-frequency speed controller, the pump power supply, and the electromagnetic flowmeter power supply. Adjust the pump variable-frequency speed controller to the calibrated value from the preliminary trial.
- After 30 minutes of operation, check whether the two conditions are still met: a 25–26 cm water level difference (hydraulic head) between the most downstream chamber and the weir crest, and an electromagnetic flowmeter reading of 8 L/s. If they are, proceed to the next step. If a slight deviation is observed, slowly add water to the most downstream chamber while adjusting the pump frequency to meet the experimental requirements. If a significant change is noted, recalibrate the experimental conditions by following the preliminary trial procedure.
- Start the video recording equipment, introduce the test specimens (Acrossocheilus fasciatus), and have all personnel move away from the model. The experiment duration is 2.5 hours.
- Since smaller fish are more difficult to catch within the model, the testing order proceeds from larger to smaller size groups: >13 cm, 11–13 cm, 9–11 cm, and <9 cm. For the first formal experiment, a total of 640 test fish were used across two independent biological replicates, with 320 fish per replicate (divided into 4 body length groups of 80 fish each). After a 2-hour observation period for one group (total trial duration: 2.5 hours, including a 30-minute acclimation period), the fish are captured from the model using a net and temporarily held elsewhere. The next size group is then introduced, and this process is repeated until all 8 groups are completed.
- Perform a preliminary analysis of the experimental data, then conduct a second formal experiment following the aforementioned procedure.

**Step 3: Data Analysis**

- Extract the leaping trajectories of the specimens from the video recordings and perform statistical analysis.

**4.Fish Acclimatization and Injury Prevention Strategy for *Acrossocheilus fasciatus* Leaping Experiments**

During the experimental study on the leaping ability of Acrossocheilus fasciatusover low weirs, our research team strictly adhered to animal ethics guidelines, with minimal intervention as the core principle. A systematic strategy for fish acclimatization and injury prevention was developed and implemented to ensure a balance between scientific rigor and animal welfare. The specific measures are outlined below:

**Sample Collection**

The Acrossocheilus fasciatussamples used in this experiment were collected from the Majinxi River basin in Kaihua County, located in the upper reaches of the Qiantang River. All collection procedures were conducted in compliance with animal ethics guidelines and were approved by the Majin Town Government of Kaihua County. To minimize harm and stress to the fish, the collection was carried out by experienced local fishermen using fish-friendly trap cages. Following capture, the fish were immediately transferred to oxygenated transport tanks and promptly delivered to the laboratory for standardized acclimatization in holding tanks.

**Acclimatization Process​**

- After collection, the fish were immediately transferred to an indoor recirculating aquaculture system with water quality conditions (temperature, pH, dissolved oxygen, hardness, etc.) closely matching those of the original habitat. An acclimatization period of no less than 14 days was conducted.
- Acclimatization and Feeding: After collection, the fish were immediately transferred to an indoor recirculating aquaculture system with water quality conditions (temperature, pH, dissolved oxygen, hardness, etc.) closely matching those of the original habitat. An acclimatization period of no less than 14 days was conducted. During this period, the fish were fed once daily at scheduled times. The diet consisted of commercial sinking algal pellets, supplemented with frozen copepods, to mimic their natural food sources. The daily feeding ration was controlled at 2-3% of the total fish body weight, and any residual feed was promptly removed to minimize water quality deterioration.​​
- Hydrodynamic Adaptation: To reduce stress and acclimatize them to the experimental conditions, a progressive flow velocity training regimen was implemented. Using a circulating water pump, a directional flow was created within the holding tank. The flow velocity was gradually increased from static conditions (0 m/s) at the beginning to the target experimental velocity of 0.4 m/s over the two-week period. This procedure ensured the fish were fully acclimated to the hydrodynamic environment prior to the formal experiments.
- Feeding was carried out with diets resembling their natural food sources, provided regularly and quantitatively each day to ensure the fish remained in healthy condition throughout the experiment.

**Rearing Conditions**

- Rearing setup: A recirculating filtration tank (dimensions: 2 m × 2 m × 1.5 m) was used, as shown in Fig4. Batch trials were conducted with 320 fish per batch, maintaining a stocking density of approximately 50 fish per cubic meter.
- Acclimation protocol: Specimens underwent a 2-week acclimation period in holding tanks after acquisition, following standardized fish physiological experiment protocols.
- Key parameters maintained:

Dechlorinated tap water (aerated for 48 hours) was used during acclimation.

Water temperature: 22°C

Dissolved oxygen: ≥7.0 mg/L

Ammonia nitrogen: <0.01 mg/L

Lighting: Well-lit ambient indoor conditions.


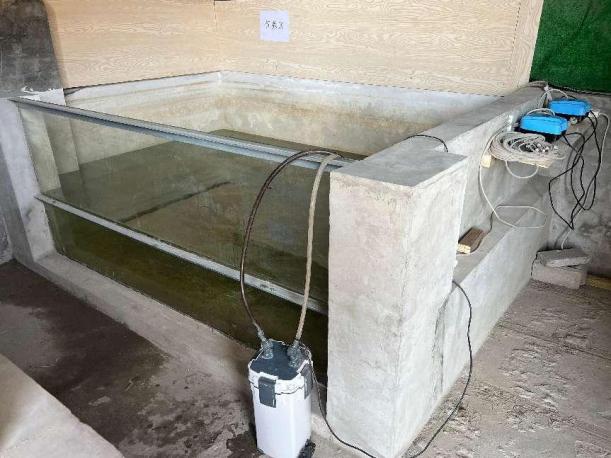
 **
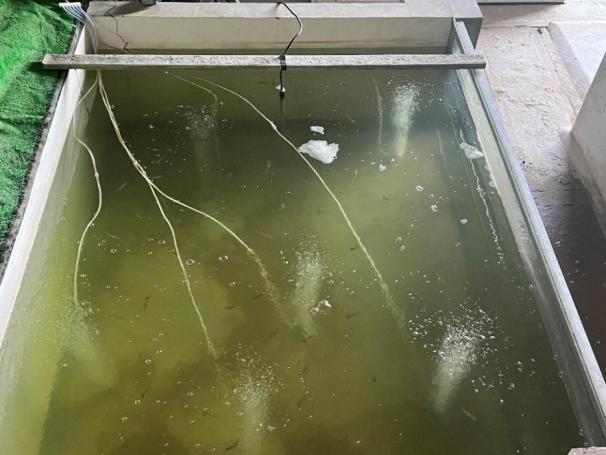
**

**Oxygenator**

**Integrated monitoring system**

**Recirculating filtration tank**

**Fig. 4 The Photo of rearing conditions**

**Environmental and Operational Injury Prevention**

- The experimental flume featured smooth inner walls and no sharp edges. Protective nets were installed at inlets and outlets to prevent physical abrasion or collision during swimming or leaping.
- All experimental operations (such as transferring, measuring, and observing) were conducted in water or under moist conditions. When necessary, soft nets were used, and air exposure was limited to ≤20 seconds per handling to minimize mucosal damage and hypoxia risks.
- No forced barriers, electrical stimuli, or chemical agents were applied during the leaping tests. The experiments relied entirely on simulated natural hydrodynamic conditions to encourage voluntary leaping behavior.

**Health Monitoring and Emergency Measures**

- Daily records were kept of fish activity, feeding behavior, and physical integrity. Any abnormal individuals were isolated immediately and excluded from further testing.
- The aquaculture system was equipped with real-time water quality monitoring and automated purification devices to ensure parameters such as ammonia and nitrite remained below safe thresholds.
- A first-aid kit for fish, including anti-stress agents and mild antiseptics for minor injuries, was prepared. Emergency protocols were established and carried out by designated personnel.

**Post-Experimental Handling and Release**

- After the experiment, all fish underwent a recovery observation period of at least 48 hours to confirm normal swimming, feeding, and absence of injuries.
- Prior to release, water conditions were gradually adjusted to match those of the original collection site. Release was conducted during periods of stable weather and gentle flow at or near the original collection point to ensure smooth reintegration into the natural population.

This strategy was applied throughout the experimental cycle to minimize the impact of human intervention on fish physiology and behavior, ensuring both the validity of the research outcomes and ecological ethics. Our research team remains committed to optimizing these procedures and welcomes oversight from peers and ethics committees.


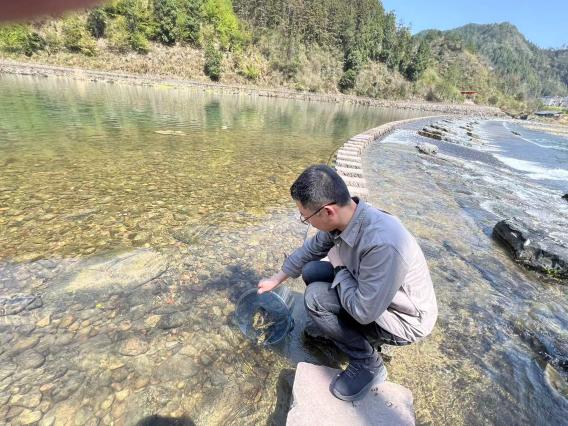

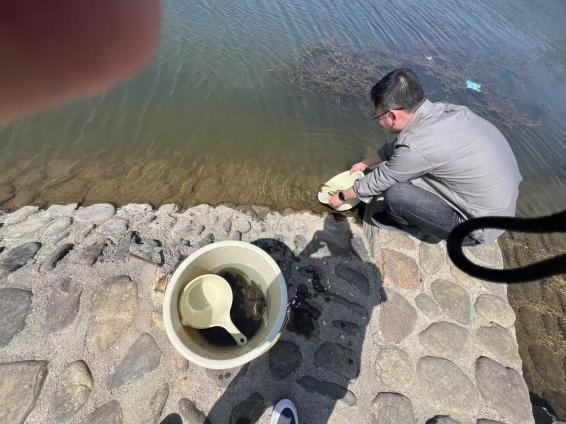


**Fig. 5 Photos of released fish after the experiment was completed**

**5.Statistical Analysis**

To address the non-independence of data arising from testing unmarked fish in groups, all quantitative statistical analyses in this study were performed at the level of the "independent trial group". The experiment comprised 8 independent trial groups (4 body length classes: <9 cm, 9-11 cm, 11-13 cm, >13 cm × 2 biological replicates). For each trial group, the following aggregated metrics were calculated from the video-analyzed behavioral data (see Section 5, "Experimental data statistics"), with each aggregated metric treated as a single, independent data point for subsequent inferential statistics:

- Passage Success Rate (PSR): The percentage of fish (out of the 80 individuals introduced per trial) that successfully passed the first weir stage.
- Proportion of Passage Strategy Usage: The proportion of successful individuals within a trial that employed the Pure Leaping Strategy (PLS) versus the Leaping-Swimming Strategy (LSS).
- Median Kinematic Parameters: The median values of takeoff position, leap distance, and leap height calculated from all successful leap events recorded within that specific trial group.

In the final manuscript, all between-group comparisons (e.g., performance across body length classes) and correlation analyses (e.g., the relationship between body length and leap distance) were based exclusively on these 8 aggregated data points​ derived from the trial groups. The raw, individual-level data presented in Section 5 of this document served as the foundation for calculating these group-level summaries. These raw data are retained to ensure transparency and reproducibility but were not used directly for inferential statistical testing in the main paper to avoid pseudoreplication.

The raw data at the individual level were used only to calculate summary metrics at the experimental group level and were not directly used for inferential statistical analysis to avoid the issue of pseudoreplication.

**6.Experimental data statistics**

The following data are based on the statistics of the experimental videos.

**Table 1 Summary Table of Experimental Parameters for Successful Upstream Passage Across Body Length Classes**

| **Table 1-1: >13 cm Body Length Group** | | | | |
| --- | --- | --- | --- | --- |
| Group 1 | | | | |
| No. | Takeoff Position(cm) | Leap Distance(cm) | Leap Height(cm) | Passage strategy |
| 1 | 6 | 1 | 20 | Leaping-Swimming Strategy |
| 2 | 28 | 33 | 45 | Pure Leaping Strategy |
| 3 | 6 | 1 | 14 | Leaping-Swimming Strategy |
| 4 | 26 | 36 | 48 | Pure Leaping Strategy |
| 5 | 10 | 30 | 37 | Pure Leaping Strategy |
| 6 | 7 | 6 | 22 | Leaping-Swimming Strategy |
| 7 | 22 | 20 | 37 | Pure Leaping Strategy |
| 8 | 7 | 2 | 13 | Leaping-Swimming Strategy |
| 9 | 12 | 27 | 35 | Pure Leaping Strategy |
| 10 | 10 | 25 | 35 | Pure Leaping Strategy |
| 11 | 25 | 35 | 30 | Pure Leaping Strategy |
| 12 | 15 | 22 | 35 | Pure Leaping Strategy |
| Average | 14.5 | 19.8 | 30.9 |  |
| Group 2 | | | | |
| No. | Takeoff Position(cm) | Leap Distance(cm) | Leap Height(cm) | Passage strategy |
| 1 | 7 | 1 | 15 | Leaping-Swimming Strategy |
| 2 | 8 | 3 | 12 | Leaping-Swimming Strategy |
| 3 | 6 | 5 | 18 | Leaping-Swimming Strategy |
| 4 | 16 | 9 | 35 | Pure Leaping Strategy |
| 5 | 6 | 5 | 23 | Leaping-Swimming Strategy |
| 6 | 8 | 2 | 16 | Leaping-Swimming Strategy |
| 7 | 13 | 28 | 37 | Pure Leaping Strategy |
| 8 | 14 | 24 | 25 | Pure Leaping Strategy |
| 9 | 6 | 4 | 17 | Leaping-Swimming Strategy |
| 10 | 17 | 27 | 30 | Pure Leaping Strategy |
| 11 | 6 | 5 | 23 | Leaping-Swimming Strategy |
| 12 | 22 | 30 | 43 | Pure Leaping Strategy |
| 13 | 28 | 28 | 32 | Pure Leaping Strategy |
| 14 | 25 | 34 | 48 | Pure Leaping Strategy |
| 15 | 12 | 27 | 35 | Pure Leaping Strategy |
| 16 | 13 | 28 | 35 | Pure Leaping Strategy |
| 17 | 12 | 27 | 26 | Pure Leaping Strategy |
| 18 | 12 | 27 | 25 | Pure Leaping Strategy |
| Average | 12.8 | 17.4 | 27.5 |  |

| **Table 1-2: 11–13 cm Body Length Group** | | | | |
| --- | --- | --- | --- | --- |
| Group 1 | | | | |
| No. | Takeoff Position(cm) | Leap Distance(cm) | Leap Height(cm) | Passage strategy |
| 1 | 15 | 30 | 45 | Pure Leaping Strategy |
| 2 | 10 | 13 | 53 | Pure Leaping Strategy |
| 3 | 7 | 6 | 18 | Leaping-Swimming Strategy |
| 4 | 5 | 4.5 | 20 | Leaping-Swimming Strategy |
| 5 | 13 | 18 | 30 | Pure Leaping Strategy |
| 6 | 7 | 5 | 22 | Leaping-Swimming Strategy |
| 7 | 14 | 24 | 25 | Pure Leaping Strategy |
| 8 | 6 | 4 | 18 | Leaping-Swimming Strategy |
| 9 | 6 | 6 | 16 | Leaping-Swimming Strategy |
| 10 | 13 | 18 | 35 | Pure Leaping Strategy |
| 11 | 15 | 30 | 52 | Pure Leaping Strategy |
| 12 | 7 | 3 | 25 | Leaping-Swimming Strategy |
| 13 | 15 | 30 | 45 | Pure Leaping Strategy |
| 14 | 6 | 3 | 20 | Leaping-Swimming Strategy |
| 15 | 14 | 24 | 25 | Pure Leaping Strategy |
| 16 | 7 | 4 | 13 | Leaping-Swimming Strategy |
| 17 | 6 | 6 | 21 | Leaping-Swimming Strategy |
| 18 | 16 | 26 | 55 | Pure Leaping Strategy |
| 19 | 10 | 15 | 28 | Pure Leaping Strategy |
| Average | 10.1 | 14.2 | 29.8 |  |
| Group 2 | | | | |
| No. | Takeoff Position(cm) | Leap Distance(cm) | Leap Height(cm) | Passage strategy |
| 1 | 13 | 18 | 35 | Pure Leaping Strategy |
| 2 | 13 | 18 | 30 | Pure Leaping Strategy |
| 3 | 15 | 25 | 54 | Pure Leaping Strategy |
| 4 | 15 | 30 | 52 | Pure Leaping Strategy |
| 5 | 8 | 4 | 23 | Leaping-Swimming Strategy |
| 6 | 4 | 3.5 | 20 | Leaping-Swimming Strategy |
| 7 | 7 | 6 | 17 | Leaping-Swimming Strategy |
| 8 | 14 | 23 | 27 | Pure Leaping Strategy |
| 9 | 7 | 7 | 18 | Leaping-Swimming Strategy |
| 10 | 15 | 30 | 45 | Pure Leaping Strategy |
| 11 | 13 | 18 | 30 | Pure Leaping Strategy |
| 12 | 15 | 25 | 55 | Pure Leaping Strategy |
| 13 | 8.5 | 5 | 18 | Leaping-Swimming Strategy |
| 14 | 7 | 6.5 | 23 | Leaping-Swimming Strategy |
| 15 | 8.5 | 16.5 | 27 | Pure Leaping Strategy |
| 16 | 10 | 13 | 53 | Pure Leaping Strategy |
| Average | 10.8 | 15.5 | 32.9 |  |

| **Table 1-3: 9–11 cm Body Length Group** | | | | |
| --- | --- | --- | --- | --- |
| Group 1 | | | | |
| No. | Takeoff Position(cm) | Leap Distance(cm) | Leap Height(cm) | Passage strategy |
| 1 | 6.5 | 16.5 | 24 | Pure Leaping Strategy |
| 2 | 4.5 | 2.5 | 20 | Leaping-Swimming Strategy |
| 3 | 4.5 | 4 | 18 | Leaping-Swimming Strategy |
| 4 | 8 | 18 | 35 | Pure Leaping Strategy |
| 5 | 5 | 3 | 16 | Leaping-Swimming Strategy |
| 6 | 4.5 | 2.5 | 19 | Leaping-Swimming Strategy |
| 7 | 10 | 15 | 24 | Pure Leaping Strategy |
| 8 | 12 | 27 | 26 | Pure Leaping Strategy |
| 9 | 6.5 | 3 | 20 | Leaping-Swimming Strategy |
| 10 | 13 | 23 | 40 | Pure Leaping Strategy |
| 11 | 4.5 | 2.5 | 18 | Leaping-Swimming Strategy |
| 12 | 12 | 17 | 40 | Pure Leaping Strategy |
| 13 | 5.5 | 4 | 16 | Leaping-Swimming Strategy |
| 14 | 4.5 | 2.5 | 13 | Leaping-Swimming Strategy |
| 15 | 5.5 | 5 | 22 | Leaping-Swimming Strategy |
| 16 | 4.5 | 2.5 | 20 | Leaping-Swimming Strategy |
| 17 | 12 | 22 | 35 | Pure Leaping Strategy |
| 18 | 4.5 | 2.5 | 16 | Leaping-Swimming Strategy |
| Average | 7.1 | 9.6 | 23.4 |  |
| Group 2 | | | | |
| No. | Takeoff Position(cm) | Leap Distance(cm) | Leap Height(cm) | Passage strategy |
| 1 | 6.5 | 5 | 20 | Leaping-Swimming Strategy |
| 2 | 4.5 | 2.5 | 17 | Leaping-Swimming Strategy |
| 3 | 6.5 | 4 | 22 | Leaping-Swimming Strategy |
| 4 | 5.5 | 3 | 22 | Leaping-Swimming Strategy |
| 5 | 4.5 | 2.5 | 15 | Leaping-Swimming Strategy |
| 6 | 4.5 | 2.5 | 19 | Leaping-Swimming Strategy |
| 7 | 6.5 | 4.5 | 21 | Leaping-Swimming Strategy |
| 8 | 9 | 14 | 24 | Pure Leaping Strategy |
| 9 | 14 | 14 | 30 | Pure Leaping Strategy |
| 10 | 6.5 | 3 | 22 | Leaping-Swimming Strategy |
| 11 | 11 | 16 | 27 | Pure Leaping Strategy |
| 12 | 10 | 15 | 24 | Pure Leaping Strategy |
| 13 | 6.5 | 6.5 | 23 | Leaping-Swimming Strategy |
| 14 | 6.5 | 6 | 24 | Leaping-Swimming Strategy |
| 15 | 4.5 | 2.5 | 19 | Leaping-Swimming Strategy |
| 16 | 12 | 15.5 | 26 | Pure Leaping Strategy |
| 17 | 4.5 | 2.5 | 22 | Leaping-Swimming Strategy |
| Average | 7.2 | 7.0 | 22.2 |  |

| **Table 1-4: <9 cm Body Length Group** | | | | |
| --- | --- | --- | --- | --- |
| Group 1 | | | | |
| No. | Takeoff Position(cm) | Leap Distance(cm) | Leap Height(cm) | Passage strategy |
| 1 | 7 | 6 | 20 | Leaping-Swimming Strategy |
| 2 | 12 | 17 | 40 | Pure Leaping Strategy |
| 3 | 6 | 5 | 19 | Leaping-Swimming Strategy |
| 4 | 10 | 18 | 30 | Pure Leaping Strategy |
| 5 | 6 | 5 | 24 | Leaping-Swimming Strategy |
| 6 | 10 | 20 | 30 | Pure Leaping Strategy |
| 7 | 7 | 6 | 16 | Leaping-Swimming Strategy |
| 8 | 6 | 3 | 19 | Leaping-Swimming Strategy |
| 9 | 5 | 4.5 | 18 | Leaping-Swimming Strategy |
| 10 | 6 | 3 | 22 | Leaping-Swimming Strategy |
| Average | 7.5 | 8.8 | 23.8 |  |
| Group 2 | | | | |
| No. | Takeoff Position(cm) | Leap Distance(cm) | Leap Height(cm) | Passage strategy |
| 1 | 6 | 5 | 16 | Leaping-Swimming Strategy |
| 2 | 16 | 26 | 30 | Pure Leaping Strategy |
| 3 | 4.5 | 4 | 20 | Leaping-Swimming Strategy |
| 4 | 10 | 15 | 35 | Pure Leaping Strategy |
| 5 | 6 | 5 | 19 | Leaping-Swimming Strategy |
| 6 | 11 | 20 | 45 | Pure Leaping Strategy |
| 7 | 7 | 5 | 22 | Leaping-Swimming Strategy |
| 8 | 10 | 15 | 32 | Pure Leaping Strategy |
| 9 | 6 | 6 | 22 | Leaping-Swimming Strategy |
| 10 | 5 | 5 | 19 | Leaping-Swimming Strategy |
| 11 | 6 | 5 | 20 | Leaping-Swimming Strategy |
| 12 | 4.5 | 2.5 | 13 | Leaping-Swimming Strategy |
| Average | 7.7 | 9.5 | 24.4 |  |

**Data for Figure 5**

| Mean Body Length(cm) | | 8 | 10 | 12 | 14 |
| --- | --- | --- | --- | --- | --- |
| Percentage Employing PLS per Trial Group (%) | Group 1 | 30.00 | 29.41 | 52.63 | 61.11 |
|  | Group 2 | 33.33 | 38.89 | 62.50 | 66.67 |

**Data for Figure 6b**

| Mean Body Length(cm) | | 8 | 10 | 12 | 14 |
| --- | --- | --- | --- | --- | --- |
| Mean Takeoff Position per Trial Group(cm) | Group 1 | 7.5 | 7.08 | 10.11 | 14.5 |
|  | Group 2 | 7.67 | 7.24 | 10.81 | 12.83 |

**Data for Figure 6d**

| Mean Body Length(cm) | | 8 | 10 | 12 | 14 |
| --- | --- | --- | --- | --- | --- |
| Mean Leap Distance per Trial Group(cm) | Group 1 | 8.75 | 9.58 | 14.18 | 19.83 |
|  | Group 2 | 9.46 | 7.00 | 15.53 | 17.44 |

**Data for Figure 6f**

| Mean Body Length(cm) | | 8 | 10 | 12 | 14 |
| --- | --- | --- | --- | --- | --- |
| Mean Leap Height per Trial Group(cm) | Group 1 | 23.80 | 23.44 | 29.79 | 30.92 |
|  | Group 2 | 24.42 | 22.18 | 32.94 | 27.50 |

**Figure 6a, 6c, and 6f are based on the data from Table 1.**
